# The transcription factor NRF2 enhances melanoma malignancy by blocking differentiation and inducing COX2 expression

**DOI:** 10.1038/s41388-020-01477-8

**Published:** 2020-09-25

**Authors:** Christina Jessen, Julia K. C. Kreß, Apoorva Baluapuri, Anita Hufnagel, Werner Schmitz, Susanne Kneitz, Sabine Roth, André Marquardt, Silke Appenzeller, Carsten P. Ade, Valerie Glutsch, Marion Wobser, José Pedro Friedmann-Angeli, Laura Mosteo, Colin R. Goding, Bastian Schilling, Eva Geissinger, Elmar Wolf, Svenja Meierjohann

**Affiliations:** 1 Department of Physiological Chemistry, University of Würzburg, Würzburg, Germany; 2 Institute of Pathology, University of Würzburg, Würzburg, Germany; 3 Department of Biochemistry and Molecular Biology, University of Würzburg, Würzburg, Germany; 4 Comprehensive Cancer Center Mainfranken, University of Würzburg, Würzburg, Germany; 5 Department of Dermatology, Venereology, and Allergology and Skin Cancer Center, University Hospital Würzburg, Würzburg, Germany; 6 Rudolf-Virchow Center for Integrative and Translational Bioimaging, University of Würzburg, Würzburg, Germany; 7 Ludwig Institute for Cancer Research, Nuffield Department of Medicine, University of Oxford, Oxford, UK

**Keywords:** Oncogenes, Melanoma

## Abstract

The transcription factor NRF2 is the major mediator of oxidative stress responses and is closely connected to therapy resistance in tumors harboring activating mutations in the NRF2 pathway. In melanoma, such mutations are rare, and it is unclear to what extent melanomas rely on NRF2. Here we show that NRF2 suppresses the activity of the melanocyte lineage marker MITF in melanoma, thereby reducing the expression of pigmentation markers. Intriguingly, we furthermore identified NRF2 as key regulator of immune-modulating genes, linking oxidative stress with the induction of cyclooxygenase 2 (COX2) in an ATF4-dependent manner. COX2 is critical for the secretion of prostaglandin E2 and was strongly induced by H_2_O_2_ or TNFα only in presence of NRF2. Induction of MITF and depletion of COX2 and PGE2 were also observed in NRF2-deleted melanoma cells in vivo. Furthermore, genes corresponding to the innate immune response such as *RSAD2* and *IFIH1* were strongly elevated in absence of NRF2 and coincided with immune evasion parameters in human melanoma datasets. Even in vitro, NRF2 activation or prostaglandin E2 supplementation blunted the induction of the innate immune response in melanoma cells. Transcriptome analyses from lung adenocarcinomas indicate that the observed link between NRF2 and the innate immune response is not restricted to melanoma.

## Introduction

Cutaneous melanoma is a tumor entity with the ability to quickly adapt to various stressors. Due to their anatomic location and biology, melanomas and their non-malignant precursors, the melanocytes, encounter numerous sources of stress. During tumor development, cutaneous melanomas are exposed to UV irradiation, causing DNA damage, oxidative stress [1, 2], and a high mutational load [3]. Although the pigment melanin, produced in melanocytes and melanomas, serves to shield the skin from UV, it is itself a source for reactive oxygen species (ROS) [4, 5]. In addition, the melanoma oncogenes BRAF or NRAS [6, 7], as well as hypoxia, nutrient deprivation, or immune mediators are common stress triggers during melanoma development and maintenance [8–11].

Adaptation to these constant threats requires efficient stress responses, which allow tumors to survive under the new, often hostile, conditions. The major transcription factor responding to ROS-induced stress is Nuclear Factor Erythroid 2-Related Factor 2 (NFE2L2 or NRF2). Due to its role in the induction of genes involved in resolving oxidative and electrophilic damage, it is known as master regulator of the oxidative stress response [12]. NRF2 has a half-life of 15–20 min and is quickly degraded in the cytosol, where it is bound to the adapter protein Kelch-like ECH-Associated Protein 1 (KEAP1). KEAP1 recruits the E3 ubiquitin ligase cullin 3 (CUL3), leading to proteasomal degradation of NRF2. Under oxidative stress conditions, several cysteine residues of KEAP1 are oxidized, resulting in dissociation from NRF2 and nuclear translocation of NRF2. Here it can bind to dimerization partners such as small MAF proteins and induce the transcription of target genes [12]. NRF2 is considered an oncogene, and activation of NRF2 by deleterious mutations of KEAP1 are found in several tumor types, such as non-small cell lung cancer (NSCLC) with 15–20% mutation frequency [13] (www.cbioportal.org). Mutational activation of the KEAP1/NRF2 pathway is connected to therapy resistance in NSCLC [14, 15]. In melanoma, the role of NRF2 is much less understood. Here, KEAP1 and NRF2 mutations are uncommon, although melanomas upregulate several antioxidant pathways including peroxiredoxins, the cysteine/glutathione pathway or NADPH regeneration [16–19]. Still, nuclear NRF2 expression, as measured by immunohistochemistry, correlates with worse overall survival in melanoma patients [20]. The recent observation that NRF2 is involved in transcriptional activation of PD-L1 [21] implies that NRF2 might play a larger role in melanoma than previously anticipated.

In this work, we set out to investigate the contribution of NRF2 for melanoma growth. The detailed characterization of global gene expression profiles and biological processes affected by NRF2 repression in vitro and in vivo allowed us to uncover an important role of NRF2 in tumor growth and immune evasion by regulating the MITF/differentiation as well as the ATF4/COX2 pathways.

## Results

### NRF2 is activated in melanoma due to cell-autonomous and non-cell autonomous effectors

**Fig. 1:**
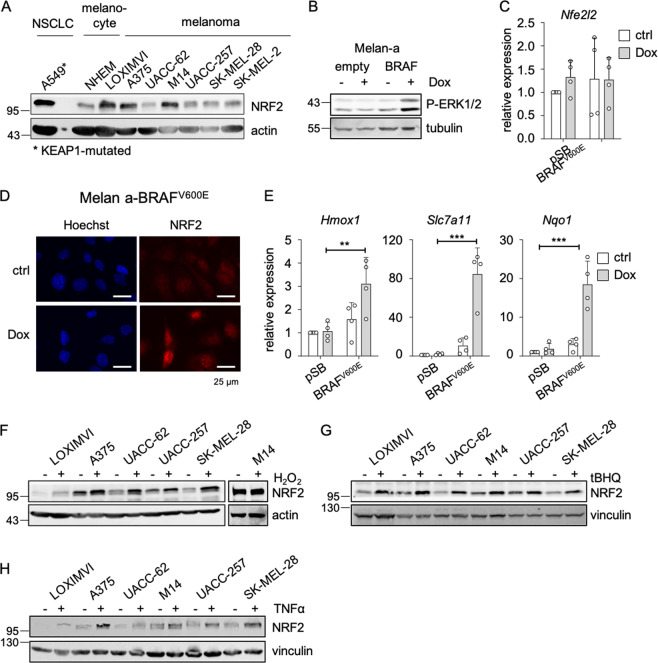
NRF2 activation in melanoma. **a** Immunoblot showing NRF2 expression in the KEAP1-mutated A549 lung adenocarcinoma cells, normal human epidermal melanocytes (NHEM) as well as indicated human melanoma cell lines. Actin served as loading control. **b** Immunoblot of P-ERK (Thr202/Tyr204) expression after Dox-dependent BRAF^V600E^ induction in the murine melanocyte cell line melan-a (100 ng/ml, 24 h). Tubulin served as a loading control. **c** Real-time PCR of *Nfe2l2* in BRAF^V600E^-expressing melan-a cells after Dox treatment (100 ng/ml, 24 h). **d** Corresponding immunofluorescence of NRF2 and Hoechst staining after BRAF^V600E^ induction. **e** Corresponding real-time PCRs of NRF2 target genes after Dox treatment (100 ng/ml, 24 h). Real-time PCR experiments were performed in three or four replicates and two-way ANOVA with Tukey’s multiple comparisons posttest was carried out (**p < 0.01, ***p < 0.001). Error bars represent SD. **f–h** Immunoblot of NRF2 in indicated melanoma cells in response to H_2_O_2_ treatment (400 µM, 5 h) (**f**), tBHQ treatment (10 µM, 24 h) (**g**), and TNFα treatment (50 ng/ml, 24 h) (**h**). Vinculin and actin served as loading controls. Please note that NRF2 is visible as singe band or double band, depending on the gel density.

To determine the level of basal NRF2 protein expression in melanoma, we performed western blots using seven melanoma cell lines, a primary neonatal human epidermal melanocyte (NHEM) cell line and the KEAP1-mutated A549 NSCLC cell line as positive control (Fig. 1a). Basal NRF2 levels were detected in all cases at varying degree. As BRAF^V600E^ is the most frequent mutation in melanoma [3, 22, 23], and BRAF and RAS oncogenes were described as potential inducers of NRF2 [24], we tested the effect of BRAF^V600E^ expression on NRF2 in melanocytes. Doxycycline (Dox)-inducible BRAF^V600E^ expression caused the expected increase in ERK1/2 phosphorylation (Fig. 1b). *Nfe2l2* RNA levels were unaffected, but an enhanced nuclear localization of NRF2 was detected after BRAF^V600E^ induction (Fig. 1c, d). This went along with a significant upregulation of the NRF2 target genes *Hmox1*, *Slc7a11*, and *Nqo1* (Fig. 1e). Next to these cell-autonomous effects, it is likely that signals from the tumor niche affect NRF2. Oxidative stress, a known inducer of NRF2 stability, is locally elevated in tumors under hypoxic conditions or as a result of macrophage infiltration [25]. After exposition of melanoma cell lines to H_2_O_2_, NRF2 levels were elevated in most cases (Fig. 1f). Similar effects were observed with the NRF2 activator tert-butylhydroquinone (tBHQ) (Fig. 1g). Infiltrating macrophages or T cells also secrete cytokines such as tumor necrosis factor α (TNFα), thereby supporting an inflammatory environment [11, 26]. In all investigated cell lines, TNFα treatment enhanced NRF2 levels (Fig. 1h). Concludingly, NRF2 is activated in melanoma due to cancer-autonomous effectors as well as effectors from the tumor niche.

### Antioxidant capacity and proliferation maintenance by NRF2 in melanoma cells

**Fig. 2:**
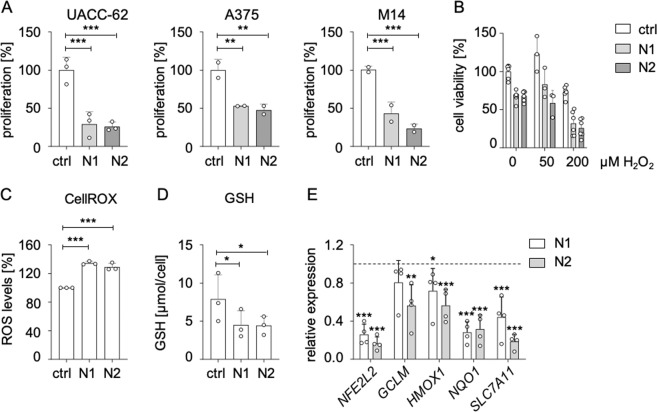
Deleterious effect of NRF2 knockdown in melanoma. **a** Proliferation of indicated melanoma cell lines in response to siRNA transfection with control siRNA or two independent *NFE2L2*-specific siRNAs. Cells were counted after 5 days. The experiments were carried out four times in duplicates (UACC-62) or two times in triplicates (A375, M14). ctrl: control siRNA; N1: *NFE2L2*-specific siRNA #1; N2: *NFE2L2*-specific siRNA #2. **b** Cell viability, as determined by MTT assay, in UACC-62 cells after transfection with control or *NFE2L2*-specific siRNA. For the last 24 h, cells were treated with H_2_O_2_. MTT was done in triplicates. **c** Intracellular ROS levels in UACC-62 cells, 3 days after transfection with control or *NFE2L2*-specific siRNA. CellROX assay was performed three times in triplicates. For **a–c**, data were normalized to the controls, which were set as 100%. **d** Intracellular glutathione concentration in UACC-62 cells three days after transfection with control or *NFE2L2*-specific siRNA (measured by Tietze assay). Assay was performed three times in duplicates. **e** Gene expression of *NFE2L2* and indicated target genes after 3 days of siRNA-mediated knockdown of *NFE2L2*. Relative expression levels referred to control siRNA are shown (dotted line). Two-tailed Student’s *t* test was carried out to calculate significant differences between each *NFE2L2-*specific siRNA and the control siRNA (*p < 0.05, **p < 0.01, ***p < 0.001). Error bars represent SD.

**Fig. 3:**
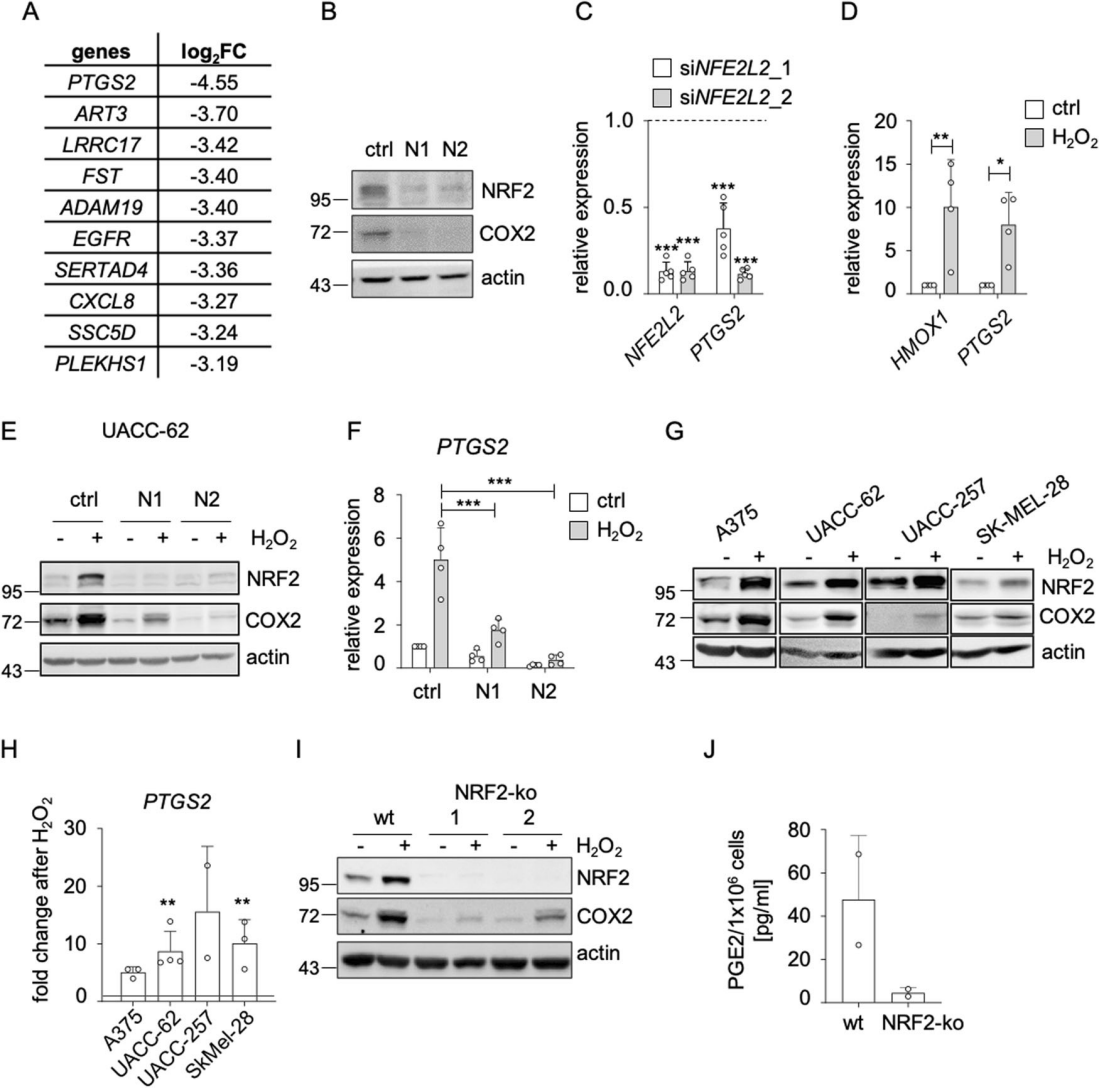
Regulation of COX2 by NRF2. **a** Table showing the ten genes with the strongest repression after siRNA mediated *NFE2L2* knockdown (N2), determined by RNA-sequencing. **b** Immunoblot showing COX2 and NRF2 expression after knockdown of *NFE2L2* (3 d) in UACC-62 cells. Actin served as loading control. **c** Corresponding real-time PCR analysis, done five times. The relative gene expression, referred to non-targeting siRNA (dotted line), is shown. **d** Real-time PCR showing expression of the NRF2 target gene *HMOX1* and *PTGS2* after H_2_O_2_ treatment in UACC-62 cells (400 µM, 5 h). The experiment was done four times. **e** Immunoblot of NRF2 and COX2 in UACC-62 cells after a combination of H_2_O_2_ treatment (400 µM, for the last 5 h of siRNA treatment) and siRNA-mediated *NFE2L2* knockdown (3 d). Actin served as loading control. **f** Corresponding real-time PCR of *PTGS2*. The experiment was done four times and two-way ANOVA with Sidak’s multiple comparisons posttest was carried out. **g** Immunoblot of NRF2 and COX2 in indicated melanoma cell lines after H_2_O_2_ treatment (400 µM, 5 h). Actin served as loading control. **h** Corresponding real-time PCR of *PTGS2*, referred to its expression in cells without treatment (dotted line). The experiment was done twice (UACC-257), three times (A375, SK-MEL-28) or four times (UACC-62). **i** Immunoblot of NRF2 and COX2 after H_2_O_2_ treatment (400 µM, 5 h) in UACC-62 control cells and in two independent UACC-62 NRF2-ko cell lines. Actin served as loading control. **j** ELISA assay for PGE2 concentration in the supernatant of UACC-62 *NFE2L2* wt and *NFE2L2* knockout cells (corresponding to NRF2-ko 1 from **I**), done in duplicates. For **c**, **d**, **h** two-tailed Student’s *t* test to calculate significant differences to the respective control was carried out (*p < 0.05, **p < 0.01, ***p < 0.001). All error bars represent SD.

To test whether melanoma cells require NRF2 for growth, we downregulated *NFE2L2* using two independent siRNAs in three different melanoma cell lines. In all cases, the knockdown led to a substantial decrease in proliferation (Fig. 2a). This effect was least pronounced in A375 cells, which showed the lowest knockdown efficiency (Supplementary Fig. 1A). *NFE2L2* knockdown furthermore resulted in an enhanced sensitivity toward oxidative stress (Fig. 2b), elevated ROS levels (Fig. 2c) and reduced intracellular glutathione content (Fig. 2d), as determined in UACC-62 cells, confirming that NRF2 is required for the maintenance of the intracellular redox balance in melanoma cells. The observed dysbalance of ROS and glutathione was accompanied by the reduction of antioxidant target genes of NRF2 (Fig. 2e). To get insight into further processes affected by NRF2, we performed RNA sequencing of UACC-62 melanoma cells transfected with control or *NFE2L2*-specific siRNA (N2). In total, 944 genes were downregulated, and 357 genes were induced by *NFE2L2* knockdown. As expected, many genes of the “Hallmark” group “ROS” were suppressed under conditions of *NFE2L2* knockdown (Supplementary Fig. 1B). Other major dysregulated gene sets including cell cycle/replication genes as well as mitochondrial genes were confirmed by real-time PCR, using both independent siRNAs (N1, N2) (Supplementary Fig. 2A–C). However, the strongest suppression was observed for genes unrelated to these gene sets (Fig. 3a).

### NRF2 mediates the expression of inducible cyclooxygenase COX2

*PTGS2* was the gene most heavily suppressed by NRF2 depletion, and we could confirm this effect by protein blot and real-time PCR with two independent siRNAs (Fig. 3b, c). *PTGS2* encodes cyclooxygenase 2 (COX2), which enables the formation of lipid mediators such as prostaglandins from phospholipid-derived arachidonic acid. COX2 was shown to elevate prostaglandin E2 (PGE2) levels, thereby fostering an immune-evasive tumor environment [27, 28]. In contrast to its counterpart COX1, which is expressed under basal conditions, COX2 expression is triggered by external factors. We observed that oxidative stress by H_2_O_2_ strongly induced the expression of *PTGS2* to levels comparable to the NRF2 target gene HMOX1 (Fig. 3d). NRF2 was responsible for the ROS-dependent induction of COX2, as NRF2-specific siRNA prevented ROS-induced COX2 expression in UACC-62 (Fig. 3e, f) as well as A375 melanoma cells (Supplementary Fig. 3A, B). The H_2_O_2_-induced COX2 expression was observed in multiple melanoma cell lines (Fig. 3g, h). To test if H_2_O_2_-dependent COX2 induction is also seen under conditions of permanent NRF2 knockout, we generated NRF2-ko UACC-62 cells using the CRISPR/Cas9 method. In contrast to acute siRNA-mediated knockdown, basal proliferation and cell cycle genes were not deregulated in the NRF2-ko cells (Supplementary Fig. 3C, D), indicating that the previously observed impairment of proliferation could be compensated during the generation of single cell clones. However, antioxidant NRF2 target genes were still suppressed under conditions of oxidative stress, thus demonstrating that their expression level is inevitably connected to NRF2 (Supplementary Fig. 3E). The same was observed for COX2, which showed reduced basal and H_2_O_2_-induced levels in NRF2-knockout cells (Fig. 3i, Supplementary Fig. 3E).

To test the influence of NRF2 on PGE2 secretion, we performed PGE2-specific ELISA using medium supernatant from NRF2-ko and NRF2 overexpressing UACC-62 cells. While NRF2 depletion led to the reduction of secreted PGE2 to almost undetectable levels (Fig. 3j), doxycycline-inducible overexpression of NRF2 enhanced PGE2 (Supplementary Fig. 3F). This was even more prominent when the NRF2 activator tBHQ was added in addition to doxycycline. However, even though NRF2 induction was sufficient to induce COX2 expression, the *PTGS2* promoter, in contrast to the promoter of the well-established NRF2 target gene *HMOX1*, was not occupied by NRF2 (Supplementary Fig. 4A), indicating that NRF2 regulates *PTGS2* in an indirect manner.

### NRF2 suppresses melanoma differentiation

**Fig. 4:**
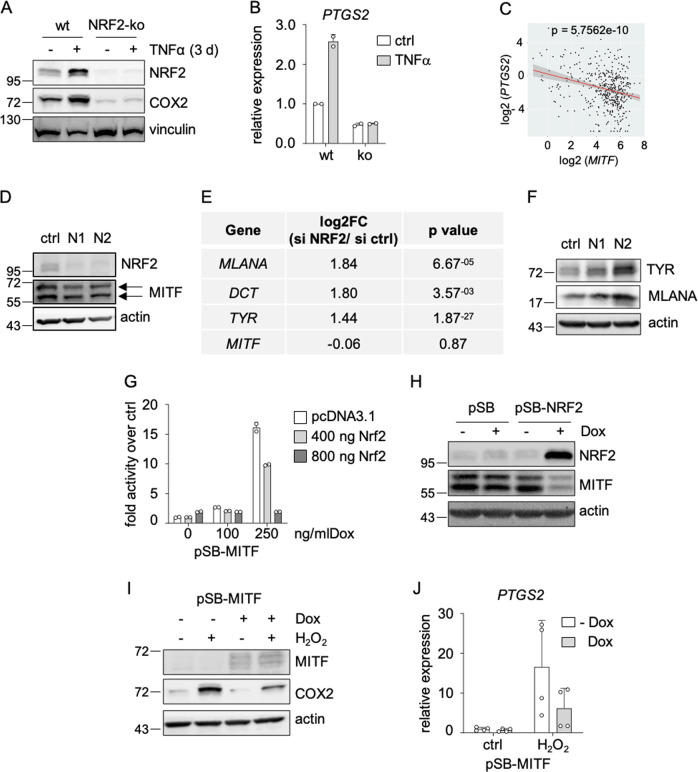
Suppression of differentiation features by NRF2. **a** Immunoblot of NRF2 and COX2 in UACC-62 *NFE2L2* wt and *NFE2L2* knockout cells after TNFα treatment (50 ng/ml, 3 d). Vinculin served as loading control. **b** Corresponding real-time PCR of *PTGS2* gene expression, derived from two independent experiments. **c** Linear regression analysis of *MITF* and *PTGS2* mRNA (*n* = 472) (Adj R2 = 0.076577 Intercept = 4.4372 Slope = −0.20477 *p* = 5.7562e-10). The results shown here are based upon data derived from the TCGA dataset Skin Cutaneous Melanoma, and FPKM values were downloaded from www.cbioportal.org. **d** Protein blot of NRF2 and MITF in UACC-62 cells after knockdown of *NFE2L2* for 3 d with two independent siRNAs. MITF is represented by both visible bands (arrows). **e** Expression changes of pigmentation genes after *NFE2L2* knockdown in UACC-62 cells, as detected by RNA sequencing, using the siRNA N2. **f** Protein blot of TYR and MLANA in UACC-62 cells after knockdown of *NFE2L2* for 3 d with two independent siRNAs. **g** Luciferase assay of UACC-62 cells after MITF induction (100 or 250 ng/ml, 3 d) and co-transfection with 400 ng or 800 ng of pcDNA3.1-NRF2 and a tyrosinase promoter construct for 2 d. Luciferase activity was measured twice in duplicates. **h** Immunoblot of NRF2 and MITF after doxycycline-dependent NRF2 induction in UACC-62 cells (1000 ng/ml Dox, 3 d). Actin served as loading control. **i** Immunoblot of MITF and COX2 in UACC-62 cells expressing the Dox-inducible MITF expression vector pSB-MITF. Where indicated, cells were treated with doxycycline (250 ng/ml, 3 d) and H_2_O_2_ was added for the last 5 h before harvesting (400 µM). **j** Corresponding real-time PCR of *PTGS2*. Data are derived from two independent experiments performed in duplicates. Error bars represent SD.

As immune-modulatory enzyme, COX2 is also induced by pro-inflammatory cytokines such as TNFα [29]. Interestingly, we found that TNFα-dependent COX2 induction was also enhanced by NRF2, as seen under different times of TNFα treatment (Fig. 4a, b: 3 days; Supplementary Fig. 4B: 12 h). In melanoma cells, TNFα causes dedifferentiation, leading to limited recognition by T cells specific for melanocytic antigens [11, 30]. Accordingly, analysis of a previously published dataset shows that *PTGS2* and *MITF* are regulated in an inverse manner after TNFα treatment in melanoma cells (Supplementary Fig. 4C) [30]. A negative correlation of *MITF* and *PTGS2* is also observed in the TCGA dataset for skin cutaneous melanomas (Fig. 4c).

Therefore, we tested if NRF2 can interfere with MITF. An *NFE2L2* knockdown had no evident effect on MITF protein levels (Fig. 4d), but resulted in the upregulation of pigmentation genes, as shown by GO analysis of the RNA sequencing data (Fig. 4e, Supplementary Fig. 5A, B). This observation was confirmed on protein level (Fig. 4f), indicating that NRF2 perturbs differentiation in melanoma. Similar observations were made in NRF2-ko UACC-62 cells, which showed an increased expression of MLANA protein (Supplementary Fig. 5C). The fact that NRF2 knockdown did not alter MITF levels, but promoted expression of its target genes MLANA, TYR and DCT, suggests that NRF2 interferes with MITF activity, but not MITF expression. Concordantly, chromatin immunoprecipitation sequencing (ChIP-Seq) did not reveal binding of NRF2 to the MITF promoter (Supplementary Fig. 5D). Also, there was no correlation between basal NRF2 levels and MITF levels in different melanoma cell lines (Supplementary Fig. 5E).

To directly assess the effect of NRF2 on MITF activity, we performed luciferase assays in UACC-62 cells with inducible MITF expression and the previously described Tyr-luc-200 vector [31]. Transient transfection of an NRF2 expression vector diminished luciferase activity in a dose-dependent manner (Fig. 4g). Interestingly, although NRF2 knockout or knockdown did not affect MITF levels, exogenous overexpression of NRF2 resulted in reduced MITF protein amount (Fig. 4h). This suggests that a strong increase of NRF2 activity reinforces the inhibitory effect on MITF and is finally manifested in lowered MITF levels.

To further analyze the effect of acute NRF2 induction on differentiation genes, we used H_2_O_2_ and TNFα as they represent NRF2 triggers with transient (H_2_O_2_) or sustained (TNFα) effects. While H_2_O_2_ was not sufficient to cause dedifferentiation, TNFα treatment clearly reduced the expression of *MLANA*, *DCT* and *TYR* in UACC-62 and M14 cells (Supplementary Fig. 5F). To test if TNFα-induced dedifferentiation is linked to NRF2, we investigated this phenotype in NRF2-ko UACC-62 cells. We observed that TNFα-mediated repression of the differentiation genes was generally possible in NRF2-ko cells, but was less pronounced after extended times of TNFα treatment (3 d) (Supplementary Fig. 5G).

**Fig. 5:**
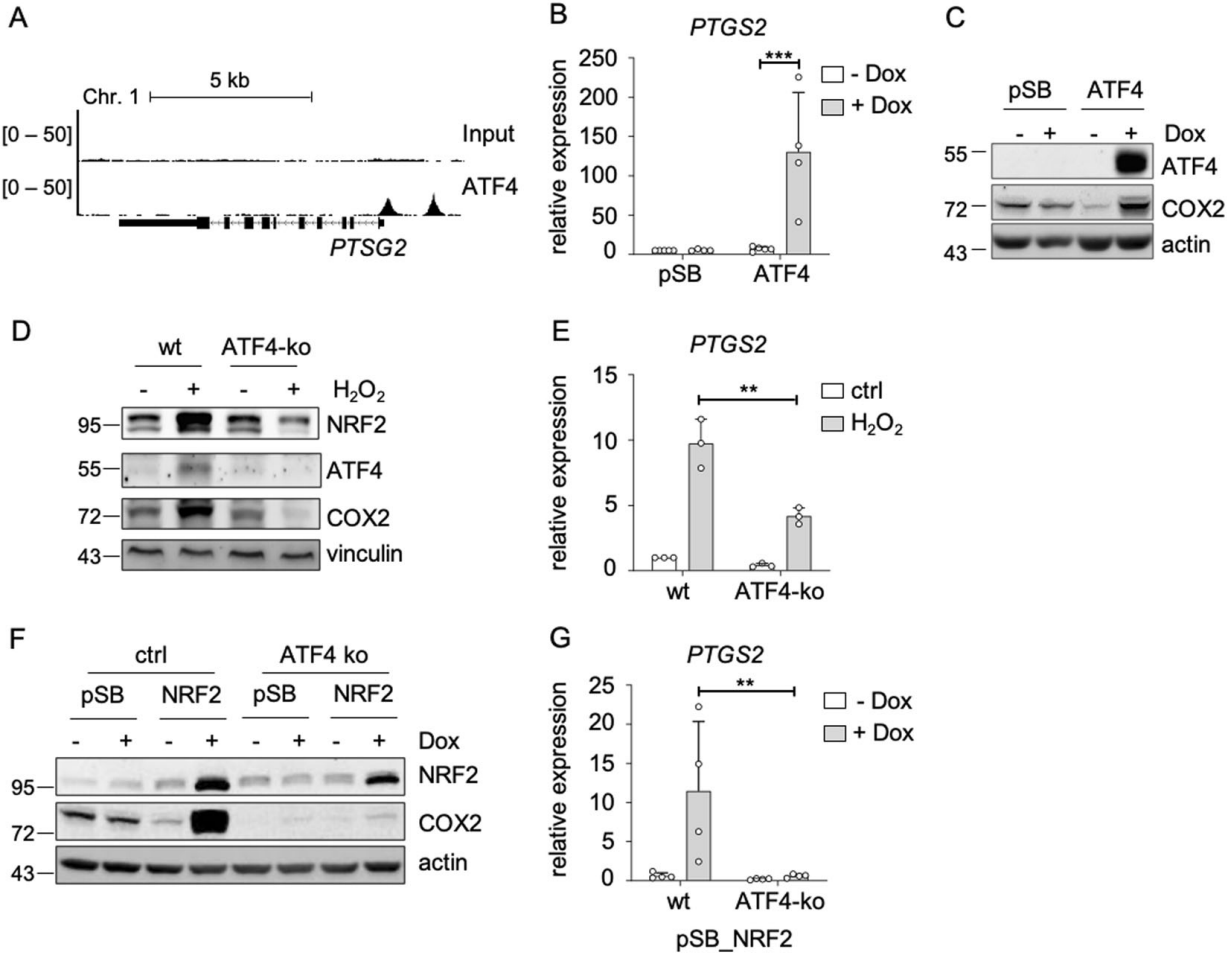
ATF4-dependent induction of *PTGS2*. **a** Genome browser tracks of promotor regions of *PTGS2* with ATF4 binding, evaluated by ATF4 ChIP-Seq analysis in human 501mel melanoma cells and an input control. **b** Real-time PCR, showing *PTGS2* expression in UACC-62 cells transfected with control vector (pSB) or an inducible ATF4 expression vector (pSB-ATF4) after incubation with 100 ng/ml doxycycline for 3 days. Data are derived from four experiments. **c** Corresponding western blot, showing ATF4 and COX2 expression. Actin served as loading control. **d** Immunoblot of NRF2, ATF4 and COX2 in UACC-62 wt and ATF4-ko cells after H_2_O_2_ treatment (400 µM, 5 h). Vinculin served as loading control. **e** Real-time PCR for *PTGS2*, using the same conditions as described in (**d**). The experiment was done three times. **f** Western blot, showing NRF2 and COX2 expression in control vector or NRF2-expressing UACC-62 cells (ctrl) and ATF4-ko cells. Where indicated, cells were stimulated with 500 ng/ml Dox for 3 days. Actin served as loading control. **g** Corresponding real-time PCR, performed four times independently. For all real-time PCRs two-way ANOVA with Tukey’s multiple comparisons posttest was carried out (**p < 0.01, ***p < 0.001). Error bars represent SD.

When analyzing publicly available RNA sequencing datasets from melanoma cell lines, we found that the knockdown of *MITF* leads to a profound induction of *PTGS2* (Supplementary Fig. 5H). Thus, we assumed that NRF2-dependent COX2 induction is connected to reduced MITF activity. Indeed, MITF overexpression reduced H_2_O_2_-triggered COX2 induction on protein and RNA level (Fig. 4i, j). However, NRF2 also affected COX2 expression in the lung adenocarcinoma cell line A549 (Supplementary Fig. 5I), which does not express MITF, indicating that the NRF2-dependent COX2 induction is not restricted to MITF-expressing cells and is therefore regulated, at least in part, by another mechanism. It was described previously that the transcription factor ATF4 is an important NRF2 interacting protein, which mediates transcription downstream of NRF2 [32]. We found that ATF4 binds to the promoter region of *PTGS2* (Fig. 5a), and that ATF4 overexpression strongly induces *PTGS2* expression on RNA and protein level (Fig. 5b, c). Interestingly, H_2_O_2_-induced *PTGS2* expression was blunted in CRISPR/Cas9 generated ATF4-ko UACC-62 cells (Fig. 5d, e), thus implying that ATF4 is required for ROS-induced *PTGS2*. Of note, the H_2_O_2_-dependent elevation of NRF2 was also decreased under conditions of ATF4 knockout, indicating that ATF4 and NRF2 may regulate each other. Next, NRF2 was overexpressed in control and ATF4-ko cells. Dox-dependent NRF2 induction led to a strong induction of COX2, but this effect was prevented when NRF2 was overexpressed in ATF4-ko UACC-62 cells (Fig. 5f, g). These data unequivocally show that NRF2-induced COX2 expression is mediated by ATF4.

### NRF2 knockout blocks melanoma development in vivo

By enhancing an undifferentiated melanoma phenotype and enabling COX2 induction, NRF2 might affect the tumor’s ability to grow in an immune-competent environment in vivo.

To test this, we used a syngeneic melanoma model with a cell line derived from the *Tyr-CreERT2*; *BRAF*^*CA*^; *Pten*^*fl*/*fl*^ model [33] that we isolated from the tumors of melanoma-bearing mice, called #781 cells. We knocked out endogenous *Nfe2l2* to gain NRF2-deficient melanoma cells, simultaneously using two gRNAs, as indicated in Supplementary Fig. 6A. NRF2 knockout cells were first tested in vitro, where we confirmed the loss of NRF2 expression as well as a reduction of basal and H_2_O_2_-inducible NRF2 target gene expression (Supplementary Fig. 6,B, C) and an enhanced sensitivity in presence of H_2_O_2_ (Supplementary Fig. 6D) in two independent sets of NRF2-wt and -ko cells.

**Fig. 6:**
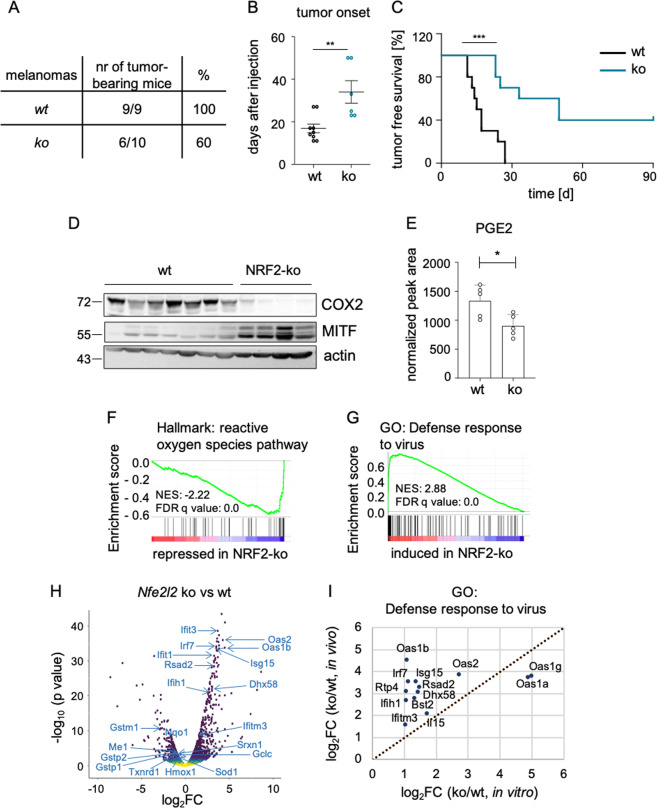
Role of NRF2 in tumor formation and immune control. **a** Table showing the number of tumor-bearing mice after subcutaneous injection of 10,000 #781 control cells (wt-1) and *Nfe2l2* knockout cells (ko-1) into the flanks of C57BL/6 mice. The experiment was stopped after 90 days. **b, c** Corresponding tumor onset (**b**) and tumor-free survival (**c**). **p < 0.01 two-tailed Student’s *t* test (**b**), ***p < 0.001, Mantel–Cox test (**c**). **d** Immunoblot of COX2 and MITF in tumors comprised of #781 control and *Nfe2l2* knockout cells excised at the experimental endpoint. Actin served as loading control. **e** Prostaglandin E2 levels in control tumors and NRF2 knockout tumors (*n* = 5 per group), measured by mass spectrometry. *p < 0.05 (two-tailed Student’s *t* test, unpaired). **f, g** GSEA enrichment plot of the Hallmark gene set “reactive oxygen species pathway” (**f**) and the Gene Ontology gene set “Defense response to virus” (**g**) in analyzed tumors. **h** Volcano plot representing deregulated genes from **f** and **g** in tumors derived from *Nfe2l2* knockout tumors versus wildtype tumors. **i** Induction of significantly regulated genes from the GO term “Defense response to virus” in *Nfe2l2* knockout cell lines versus controls and *Nfe2l2* knockout versus control tumors.

**Fig. 7:**
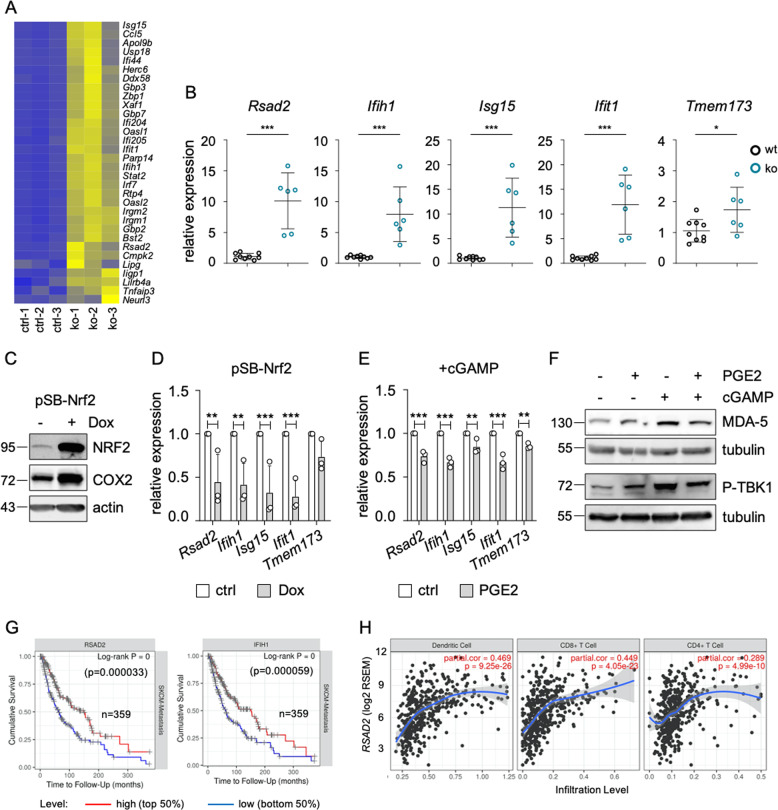
Involvement of NRF2 in innate immunity. **a** Heat plot displaying the expression of DNA sensing pathway genes, corresponding to the cGAS/STING pathway, as derived from [34], in wt (wt-1) and *Nfe2l2* knockout (ko-1) mouse tumors. **b** Expression of *Rsad2*, *Ifih1, Isg15*, *Ifit1* and *Tmem173* in control and *Nfe2l2-*deficient melanoma cells (#781). **c** Immunoblot of NRF2 and COX2 protein expression in response to Dox-inducible NRF2 expression in #781 cells (250 ng/ml, 3 d). Actin served as loading control. **d** Corresponding real-time PCR of *Rsad2*, *Ifih1, Isg15, Ifit1* and *Tmem173*, done three times. **e** Real-time PCR of *Rsad2*, *Ifih1*, *Isg15*, *Ifit1* and *Tmem173* after pathway activation by cGAMP (4 µg/ml, 4 h) and co-treatment with PGE2 (5 µM, 1 d). For **d** and **e**, two-way ANOVA with Sidak’s multiple comparisons posttest was carried out (**p < 0.01, ***p < 0.001). Error bars represent SD. **f** Immunoblot of the DNA sensing pathway proteins MDA-5 (encoded by *Ifih1*) and P-TBK1 (Ser172) after pathway activation by cGAMP (4 µg/ml, 4 h) and co-treatment with PGE2 (5 µM, 1 d). **g** Kaplan–Meier plot of cumulative survival in SKCM metastatic melanomas with high or low *RSAD2* (**left**) and *IFIH1* expression (**right**) (https://cistrome.shinyapps.io/timer/). **h** Infiltration of indicated immune cell populations into SKCM melanomas in relation to *RSAD2* expression, as determined by the Tumor Immune Estimation Resource TIMER (https://cistrome.shinyapps.io/timer/).

Next, one set of #781 control and NRF2 knockout cells were subcutaneously injected into the flanks of immunocompetent C57BL/6 mice. While all mice obtaining control melanoma cells developed tumors with a mean onset of 17 days, 40% of mice injected with NRF2 knockout cells developed no tumors at all (Fig. 6a). Those mice, in which tumors were formed, had a significantly delayed tumor onset with a mean of 34 days (Fig. 6b, c) as well as strongly decreased COX2 levels and increased MITF expression (Fig. 6d). Furthermore, PGE2 levels were reduced in NRF2-ko tumors (Fig. 6e). To get insight into differentially regulated processes of control and NRF2 knockout tumors, we performed RNA sequencing of three tumors from each group and observed a reduction of the Hallmark gene sets “ROS pathway” and “Xenobiotic metabolism”, as expected (Fig. 6f, h, Supplementary Fig. 7A). Interestingly, the most enriched elevated gene sets were all related to the immune response (Supplementary Fig. 7A). Specifically, the functional gene ontology group “Defense response to virus” was profoundly upregulated in NRF2 knockout melanomas (Fig. 6g, h). When we compared enriched gene sets in NRF2 knockout versus control tumors with those from NRF2 knockout versus control cell lines, we found that genes from this group were even more upregulated by NRF2 knockout in vivo (Fig. 6i). The gene group “Defense response to virus” is part of the innate immune response and is characteristic for the recognition of cytosolic DNA, which can be caused by viral infection but is also present in fast-growing tumor tissue. Interestingly, NRF2 knockout melanomas showed a robust elevation of a full gene set previously described to be indicative of an activated DNA sensing pathway [34] (Fig. 7a). Representative genes from this gene set such as *Rsad2*, *Ifih1*, *Isg15*, *Ifit1* and *Tmem173* were consistently found to be upregulated in all available NRF2 knockout melanomas (Fig. 7b). Conversely, when NRF2 was overexpressed in vitro, all of these genes except *Tmem173* were suppressed (Fig. 7c, d), indicating that NRF2-dependent suppression of innate immune response genes also takes place in absence of a tumor microenvironment. Furthermore, PGE2 was able to significantly blunt the acute induction of the innate immune response, caused by the STING agonist cGAMP (Fig. 7e), and reduced the phosphorylation of TBK1 and the expression of MDA-5 (encoded by *Ifih1*) (Fig. 7f).

An activated innate immune response is able to potently attract the infiltration of immune cells [35]. Accordingly, we observed the expression of *Prf1* and *Gzmb*, encoding the CD8 T cell derived cytotoxic perforin 1 and granzyme B, only in NRF2 knockout, but not in control melanomas (Supplementary Fig. 7B). In addition, the T cell markers *Cd3g* and *Cd8a* showed a trend towards higher expression in NRF2 knockout tumors (Supplementary Fig. 7B) and quantification of CD3 + immune cell infiltration into control and NRF2 knockout melanomas showed a similar trend (Supplementary Fig. 7C, D).

To assess the relevance of the innate immune response genes for human melanomas, we used RNA expression data from the TCGA SKCM metastatic melanoma dataset and analyzed the cumulative patient survival in relation to *RSAD2* and *IFIH1* expression. A high expression of both *RSAD2* and *IFIH1* was significantly associated with improved survival (Fig. 7g), in accordance with our data derived from the mouse model. We then used the Tumor IMmune Estimation Resource (TIMER) database, which allows the analysis of immune infiltrates from RNA expression data across different cancer types [36], to test the influence of *RSAD2* and *IFIH1* on immune cell infiltration. Both genes were associated with a higher infiltration of dendritic cells as well as CD4 + and CD8 + T cells (Fig. 7h, Supplementary Fig. 7E).

**Fig. 8:**
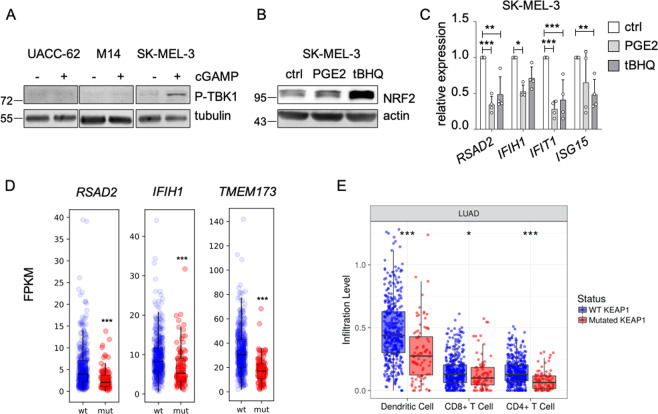
Link between innate immune response and NRF2 in human melanoma cells and KEAP1-mutant lung adenocarcinoma. **a** Immunoblot of P-TBK1 (Ser172) after pathway activation by cGAMP (4 µg/ml, 4 h) in indicated cell lines. Tubulin served as loading control. **b** Immunoblot showing NRF2 activation in SK-MEL-3 cells after stimulation with PGE2 (5 µM) or tBHQ (10 µM) for 8 h. **c** Corresponding real-time PCR of *RSAD2*, *IFIH1, ISG15* and *IFIT1*. Data are derived from four experiments. Two-way ANOVA with Tukey’s multiple comparisons posttest was carried out (**p < 0.01, ***p < 0.001). Error bars represent SD. **d** RNA expression of *RSAD2*, *IFIH1* and *TMEM173* in TCGA lung adenocarcinoma (TCGA, PanCancer Atlas), divided into KEAP1 wildtype and KEAP1 mutant tumors. Data were downloaded from the GDC portal (https://portal.gdc.cancer.gov). In case of *IFIH1*, one outlier was omitted from the plot, but was included into the boxplot calculation. **e** Immune cell infiltration into KEAP1-mutant and KEAP1-wildtype lung adenocarcinomas (LUAD) as determined by the Tumor Immune Estimation Resource TIMER (https://cistrome.shinyapps.io/timer/).

In established human melanoma cell lines, the cGAS/STING pathway is frequently dysfunctional and cannot be activated by cytosolic DNA [37]. Accordingly, cGAMP was not able to stimulate the STING pathway in UACC-62 and M14 cells, as shown by TBK1 phosphorylation, and the “Defense response to virus” gene set was not enriched in the RNA sequencing dataset from UACC-62 cells. However, the STING pathway could be activated in SK-MEL-3 cells (as reported previously [37]) (Fig. 8a). Accordingly, the addition of PGE2 and the stabilization of NRF2 by tBHQ led to a reduction of *RSAD2*, *IFIH1*, *IFIT1* and *ISG15* expression in SK-MEL-3 cells (Fig. 8c). Thus, NRF2-dependent suppression of innate immune response genes can occur in human melanoma cells with a functional cGAS/STING pathway.

If NRF2 is a general inducer of an immune-suppressive environment, tumors with consistently activated NRF2 should display signs of low immunogenicity. As 20% of lung adenocarcinomas (LUAD) harbor KEAP1 mutations and thus elevated NRF2 activity, we tested the correlation between KEAP1 mutations and immune parameters in this tumor entity. Interestingly, KEAP1 mutated LUAD have significantly lower expression levels of *RSAD2*, *IFIH1* and *TMEM173* (Fig. 8d) and a significantly decreased infiltration with dendritic cells and CD4 + as well as CD8 + T cells (Fig. 8e), showing that NRF2 pathway activation favors the generation of an immune-evasive tumor microenvironment.

## Discussion

Here we describe the critical role of NRF2 in melanoma dedifferentiation, *PTGS2* expression and the corresponding suppression of innate immune response genes.

We observed that NRF2 represses MITF activity, thereby limiting the expression of numerous pigmentation antigens such as MLANA, TYR, or DCT. However, as there was no correlation between basal NRF2 levels and MITF levels in melanoma cell lines, we conclude that NRF2-inducing stress triggers rather than basal NRF2 expression are instrumental in regulating MITF activity. The proteins encoded by MITF target genes have a strong impact on the antigenicity of melanoma cells. MART-1, which is encoded by MLANA, contains a peptide that is presented by MHC-I and efficiently recognized by T cells [38]. In addition, other pigmentation markers including TYR and DCT serve as melanoma antigens [39]. Accordingly, when melanomas are driven into dedifferentiation, they can more easily escape T cell control [11]. However, dedifferentiated melanoma cells are not only characterized by a lack of MITF, but they frequently express receptor tyrosine kinases, which might affect drug resistance and immune control [40–44]. Interestingly, we could also observe in melanoma cells that NRF2 positively regulates EGFR (Fig. 3a) and therefore strengthens the dedifferentiated phenotype by simultaneously suppressing MITF activity and inducing EGFR expression.

In addition to altering differentiation antigens, MITF affected the expression of *PTGS2*. As we have identified ATF4 as the responsible transcription factor for *PTGS2* induction downstream of NRF2, it is likely that an interaction between ATF4 and MITF regulates *PTGS2* in melanoma cells. In support of this theory, it was previously reported that MITF increases the ratio between monounsaturated and saturated fatty acids, which can negatively impact ATF4 [45]. As ATF4 also suppresses MITF levels [8], both are connected by a mutually negative regulatory loop. With the NRF2-ATF4 axis, we have now revealed a major regulatory pathway for *PTGS2* induction, which is responsible for ROS-induced as well as basal *PTGS2* expression.

COX2 is required for the generation of prostaglandins such as PGE2, which is perceived as promoter of an immune-evasive tumor microenvironment, as it acts as an inhibitor of T cell receptor signaling and thereby limits T cell activation [46]. In tumors expressing COX1 and/or COX2, immune control is hampered, as PGE2 blocks the infiltration of natural killer cells and dendritic cells, thus limiting the recognition by the immune system [28]. Previously, it was described that type I interferon signaling, which overlaps with the innate immune response, is suppressed by PGE2 in COX-proficient melanomas, whereas the genetic ablation of *Ptgs2* or *Ptgs1* and *-2* enables tumor recognition by the immune system [27]. Here, we show that PGE2 already alleviates the induction of the innate immune response in a cancer-cell autonomous manner, without the influence from the tumor microenvironment. In addition, we observed that the expression of innate immune response genes goes along with an increased infiltration of immune cells in melanoma patients and a better overall survival. Intriguingly, the link between NRF2 and suppression of the innate immune gene response was also observed in KEAP1 mutant LUAD (Fig. 8d). It was previously reported that NRF2 activation by KEAP1 is significantly associated with a T cell exclusion program [47]—a concept, which was independently confirmed by us using TIMER analysis (Fig. 8e). Thus, it is likely that NRF2 regulates immune control in several tumor entities.

A connection between NRF2 and innate immune response was previously described in mouse models for viral infections, where NRF2 suppressed STING and type I interferon response and thereby enhanced the susceptibility to Herpes infections [48, 49]. In the context of cancer, STING can induce spontaneous anti-tumor T cell activity by enhancing melanoma antigenicity [50]. Accordingly, STING agonists are explored in clinical trials as novel therapeutic strategy to turn immune-cold into immune-hot tumors and thereby control tumor growth [51].

In summary, activation of NRF2 is enabled in melanoma by intrinsic and extrinsic triggers that can be encountered in the tumor niche, such as cytokines and oxidative stress. NRF2 activation in melanoma facilitates the generation of a dedifferentiated phenotype and increases the production of COX2 and PGE2, thereby limiting the innate immune response and contributing to the generation of an immune-cold tumor environment. The link between NRF2 and immune-evasive features is also found in KEAP1-mutated LUAD, thus showing that this tumor-promoting function of NRF2 is not restricted to a single tumor entity. It is therefore likely that inhibitors of NRF2 would synergize with checkpoint inhibitor therapy in different tumor entities. Due to a lack of specific NRF2 inhibitors, such a combination is currently not feasible. However, preclinical models have demonstrated that COX inhibitors as well as STING agonists synergize with anti-PD-1 immune therapy [27, 52], and thus the targeting of NRF2 downstream markers poses therapeutic opportunities.

## Materials and methods

### Cell culture

LOXIMVI, UACC-62, M14, UACC-257 and SK-MEL-2 cells were obtained from NCI/NIH (DCTD Tumor Repository, National Cancer Institute at Frederick, Frederick, Maryland, USA). A375, SK-MEL-3, SK-MEL-28 and A-549 cells were received from ATCC. Cells have been authenticated and are regularly tested for contaminations. #781 cells were generated from tumor tissue of *Tyr-Cre*^*ERT2*^*; BRAF*^*V600E*^*; Pten*^*flox/flox*^ mice [33] and one round of subcutaneous injection into C57BL/6 mice. All tumor cell lines were cultivated in DMEM with 10% FCS (PAN biotech, Aidenbach, Germany) and 1% penicillin/streptomycin (Sigma-Aldrich, St Louis, USA) at 37 °C and 5% CO_2_. NHEM cells purchased from Promocell (Heidelberg, Germany), were maintained in Ham’s F10 containing 20% fetal calf serum, 100 nM TPA (Calbiochem/Merck, Darmstadt, Germany), 200 pM cholera toxin (Calbiochem/Merck, Darmstadt, Germany), penicillin/streptomycin, 100 μM 3-isobutyl-1-methylxanthine and ITS Premix (1:1000 BD Biosciences, Heidelberg, Germany). Murine melanocytes (‘melan-a’) were originally isolated in the laboratory of Dorothy Bennett (St George’s, University of London, London, UK) and were obtained from the Wellcome Trust Functional Genomic Cell Bank (St George’s, University of London). Where indicated, doxycycline, H_2_O_2_ (both from Sigma-Aldrich, St Louis, USA), or PGE2 (Tocris Biosciences, Bristol, UK) were added. TNFα was kindly provided by Harald Wajant (Dept. of Molecular Internal Medicine, University Hospital Würzburg). Induction of DNA sensing pathway was done by transfection of cGAMP (Sigma-Aldrich, St Louis, USA) with Lipofectamin 3000 (Invitrogen, Carlsbad, USA).

### MTT assay

Cells were seeded in triplicates in a 96-well plate at equal numbers. After indicated treatment, 5 mg/ml MTT (3-(4,5-dimethylthiazol-2-yl)-2,5-diphenyltetrazolium bromide) (Sigma-Aldrich, St Louis, USA) was added (1:5) to the cells for 2 h. Afterwards, cells were lysed with 150 µl DMSO for 20 min. Analysis of the developed formazan accumulation was performed in a microplate reader (Berthold TriStar LB 941, Berthold Technologies, Bad Wildbad, Germany) at 590 nm with a reference filter of 620 nm.

### Protein lysis and western blot

Cells were lysed in RIPA lysis buffer (50 mM Tris pH 8.0; 50 mM NaCl; 1% Nonidet-P40; 0.5% deoxycholate, 0.1% SDS; 10 μg/ml aprotinin; 10 μg/ml leupeptin; 200 μM Na_3_VO_4_; 1 mM PMSF and 100 mM NaF). Generally, 40 µg of protein was separated by 12% polyacrylamide SDS-PAGE. Proteins were transferred on Amersham^TM^ nitrocellulose membranes (GE Healthcare, Chicago, USA) and blocked with 5% BSA (Serva, Heidelberg, Germany) in TBS with 0.1% Tween-20. Primary antibody incubation was carried out overnight at 4 °C. Antibodies used for immunoblot: NRF2 (1:1000 [EP1808Y], ab62532), MLANA (1:2000, ab210546), TYR (1:2000, ab170905) from Abcam, Cambridge, UK; COX2 (1:1000, #5625), ATF4 (1:1000, #11815), MDA-5(1:1000, #5321), P-TBK1/NAK (Ser172) (1:1000, #5483), and P-ERK1/2 (Thr202/Tyr204) (1:1000, #9101) from Cell Signaling Technology, Danvers, USA. For loading controls beta-actin (1:5000, sc-47778, Santa Cruz Biotechnology, Dallas, USA), tubulin (1: 5000, T6074) or vinculin (1:10000, V9131) from Sigma-Aldrich (St. Louis, USA) were used. The MITF antibody was from C. Goding (Oxford Ludwig Institute, University of Oxford). For protein detection membranes were incubated with secondary antibodies (anti-mouse, (1:3000, 31444, Thermo Scientific, Waltham, USA) or anti-rabbit (1:10000, 170–6515, BioRad, Hercules, USA) coupled to horseradish peroxidase and visualized with the ECL detection Kit (Thermo Scientific, Waltham, USA) and a CCD system (Kodak, Rochester, USA).

### Injection of murine melanoma cells into mice

For the subcutaneous injection of murine melanoma cells into flanks of C57BL/6 mice (both sexes, aged 6–7 weeks), 10,000 cells were resuspended in 1× PBS and injected into the previously shaved skin. Cells were injected visibly through bump formation. Tumor growth was monitored and documented daily. Tumor onset was determined when tumors were clearly palpable (at ~4 mm in diameter), this marked the day until tumor-free survival. The experiment was terminated when tumor size reached 15 mm in diameter. All experiments were approved by local authorities (Government of Unterfranken) and were done in accordance with the institution’s guidelines.

Further methods can be found in the Supplementary information.

## Supplementary information

Supplementary Material

## References

[1] Cotter MA, Thomas J, Cassidy P, Robinette K, Jenkins N, Florell SR (2007). N-acetylcysteine protects melanocytes against oxidative stress/damage and delays onset of ultraviolet-induced melanoma in mice. Clin Cancer Res.

[2] Sander CS, Chang H, Hamm F, Elsner P, Thiele JJ (2004). Role of oxidative stress and the antioxidant network in cutaneous carcinogenesis. Int J Dermatol.

[3] Hodis E, Watson IR, Kryukov GV, Arold ST, Imielinski M, Theurillat JP (2012). A landscape of driver mutations in melanoma. Cell.

[4] Mitra D, Luo X, Morgan A, Wang J, Hoang MP, Lo J (2012). An ultraviolet-radiation-independent pathway to melanoma carcinogenesis in the red hair/fair skin background. Nature.

[5] Meierjohann S (2014). Oxidative stress in melanocyte senescence and melanoma transformation. Eur J Cell Biol.

[6] Corazzari M, Rapino F, Ciccosanti F, Giglio P, Antonioli M, Conti B (2015). Oncogenic BRAF induces chronic ER stress condition resulting in increased basal autophagy and apoptotic resistance of cutaneous melanoma. Cell Death Differ.

[7] Leikam C, Hufnagel A, Schartl M, Meierjohann S (2008). Oncogene activation in melanocytes links reactive oxygen to multinucleated phenotype and senescence. Oncogene.

[8] Falletta P, Sanchez-Del-Campo L, Chauhan J, Effern M, Kenyon A, Kershaw CJ (2017). Translation reprogramming is an evolutionarily conserved driver of phenotypic plasticity and therapeutic resistance in melanoma. Genes Dev.

[9] Ferguson J, Smith M, Zudaire I, Wellbrock C, Arozarena I (2017). Glucose availability controls ATF4-mediated MITF suppression to drive melanoma cell growth. Oncotarget.

[10] Widmer DS, Hoek KS, Cheng PF, Eichhoff OM, Biedermann T, Raaijmakers MIG (2013). Hypoxia contributes to melanoma heterogeneity by triggering HIF1alpha-dependent phenotype switching. J Investig Dermatol.

[11] Landsberg J, Kohlmeyer J, Renn M, Bald T, Rogava M, Cron M (2012). Melanomas resist T-cell therapy through inflammation-induced reversible dedifferentiation. Nature.

[12] Rojo de la Vega M, Chapman E, Zhang DD (2018). NRF2 and the Hallmarks of Cancer. Cancer Cell.

[13] Singh A, Misra V, Thimmulappa RK, Lee H, Ames S, Hoque MO (2006). Dysfunctional KEAP1-NRF2 interaction in non-small-cell lung cancer. PLoS Med.

[14] Frank R, Scheffler M, Merkelbach-Bruse S, Ihle MA, Kron A, Rauer M (2018). Clinical and Pathological Characteristics of KEAP1- and NFE2L2-Mutated Non-Small Cell Lung Carcinoma (NSCLC). Clin Cancer Res.

[15] Park SH, Kim JH, Ko E, Kim JY, Park MJ, Kim MJ, et al. Resistance to gefitinib and cross-resistance to irreversible EGFR-TKIs mediated by disruption of the Keap1-Nrf2 pathway in human lung cancer cells. FASEB J. 2018;fj201800011R. 10.1096/fj.201800011R 29812969

[16] Schmitt A, Schmitz W, Hufnagel A, Schartl M, Meierjohann S (2015). Peroxiredoxin 6 triggers melanoma cell growth by increasing arachidonic acid-dependent lipid signalling. Biochem J.

[17] Lokaj K, Meierjohann S, Schutz C, Teutschbein J, Schartl M, Sickmann A (2009). Quantitative differential proteome analysis in an animal model for human melanoma. J Proteome Res.

[18] Leikam C, Hufnagel A, Walz S, Kneitz S, Fekete A, Muller MJ (2014). Cystathionase mediates senescence evasion in melanocytes and melanoma cells. Oncogene.

[19] Chang YL, Gao HW, Chiang CP, Wang WM, Huang SM, Ku CF (2015). Human mitochondrial NAD(P)(+)-dependent malic enzyme participates in cutaneous melanoma progression and invasion. J Investig Dermatol.

[20] Hintsala HR, Jokinen E, Haapasaari KM, Moza M, Ristimaki A, Soini Y (2016). Nrf2/Keap1 Pathway and Expression of Oxidative Stress Lesions 8-hydroxy-2’-deoxyguanosine and Nitrotyrosine in Melanoma. Anticancer Res.

[21] Zhu B, Tang L, Chen S, Yin C, Peng S, Li X (2018). Targeting the upstream transcriptional activator of PD-L1 as an alternative strategy in melanoma therapy. Oncogene.

[22] Davies H, Bignell GR, Cox C, Stephens P, Edkins S, Clegg S (2002). Mutations of the BRAF gene in human cancer. Nature.

[23] Appenzeller S, Gesierich A, Thiem A, Hufnagel A, Jessen C, Kneitz H (2019). The identification of patient-specific mutations reveals dual pathway activation in most patients with melanoma and activated receptor tyrosine kinases in BRAF/NRAS wild-type melanomas. Cancer.

[24] DeNicola GM, Karreth FA, Humpton TJ, Gopinathan A, Wei C, Frese K (2011). Oncogene-induced Nrf2 transcription promotes ROS detoxification and tumorigenesis. Nature.

[25] Reuter S, Gupta SC, Chaturvedi MM, Aggarwal BB (2010). Oxidative stress, inflammation, and cancer: how are they linked?. Free Radic Biol Med.

[26] Qu X, Tang Y, Hua S (2018). Immunological approaches towards cancer and inflammation: a cross talk. Front Immunol.

[27] Zelenay S, van der Veen AG, Bottcher JP, Snelgrove KJ, Rogers N, Acton SE (2015). Cyclooxygenase-dependent tumor growth through evasion of immunity. Cell.

[28] Bottcher JP, Bonavita E, Chakravarty P, Blees H, Cabeza-Cabrerizo M, Sammicheli S (2018). NK cells stimulate recruitment of cDC1 into the tumor microenvironment promoting cancer immune control. Cell.

[29] Subbarayan V, Sabichi AL, Llansa N, Lippman SM, Menter DG (2001). Differential expression of cyclooxygenase-2 and its regulation by tumor necrosis factor-alpha in normal and malignant prostate cells. Cancer Res.

[30] Riesenberg S, Groetchen A, Siddaway R, Bald T, Reinhardt J, Smorra D (2015). MITF and c-Jun antagonism interconnects melanoma dedifferentiation with pro-inflammatory cytokine responsiveness and myeloid cell recruitment. Nat Commun.

[31] Schepsky A, Bruser K, Gunnarsson GJ, Goodall J, Hallsson JH, Goding CR (2006). The microphthalmia-associated transcription factor Mitf interacts with beta-catenin to determine target gene expression. Mol Cell Biol.

[32] DeNicola GM, Chen PH, Mullarky E, Sudderth JA, Hu Z, Wu D (2015). NRF2 regulates serine biosynthesis in non-small cell lung cancer. Nat Genet.

[33] Dankort D, Curley DP, Cartlidge RA, Nelson B, Karnezis AN, Damsky WE (2009). Braf(V600E) cooperates with Pten loss to induce metastatic melanoma. Nat Genet.

[34] Konno H, Chinn IK, Hong D, Orange JS, Lupski JR, Mendoza A (2018). Pro-inflammation Associated with a Gain-of-Function Mutation (R284S) in the Innate Immune Sensor STING. Cell Rep..

[35] Corrales L, McWhirter SM, Dubensky TW, Gajewski TF (2016). The host STING pathway at the interface of cancer and immunity. J Clin Investig.

[36] Li T, Fan J, Wang B, Traugh N, Chen Q, Liu JS (2017). TIMER: a web server for comprehensive analysis of tumor-infiltrating immune cells. Cancer Res.

[37] Xia T, Konno H, Barber GN (2016). Recurrent Loss of STING signaling in melanoma correlates with susceptibility to viral oncolysis. Cancer Res.

[38] Coulie PG, Brichard V, Van Pel A, Wolfel T, Schneider J, Traversari C (1994). A new gene coding for a differentiation antigen recognized by autologous cytolytic T lymphocytes on HLA-A2 melanomas. J Exp Med.

[39] Fassler M, Diem S, Mangana J, Hasan Ali O, Berner F, Bomze D (2019). Antibodies as biomarker candidates for response and survival to checkpoint inhibitors in melanoma patients. J Immunother Cancer.

[40] Tsoi J, Robert L, Paraiso K, Galvan C, Sheu KM, Lay J (2018). Multi-stage differentiation defines melanoma subtypes with differential vulnerability to drug-induced iron-dependent oxidative stress. Cancer Cell.

[41] Muller J, Krijgsman O, Tsoi J, Robert L, Hugo W, Song C (2014). Low MITF/AXL ratio predicts early resistance to multiple targeted drugs in melanoma. Nat Commun.

[42] Sun C, Wang L, Huang S, Heynen GJ, Prahallad A, Robert C (2014). Reversible and adaptive resistance to BRAF(V600E) inhibition in melanoma. Nature.

[43] Aguilera TA, Rafat M, Castellini L, Shehade H, Kariolis MS, Hui AB (2016). Reprogramming the immunological microenvironment through radiation and targeting Axl. Nat Commun.

[44] Dong ZY, Zhang JT, Liu SY, Su J, Zhang C, Xie Z (2017). EGFR mutation correlates with uninflamed phenotype and weak immunogenicity, causing impaired response to PD-1 blockade in non-small cell lung cancer. Oncoimmunology.

[45] Vivas-Garcia Y, Falletta P, Liebing J, Louphrasitthiphol P, Feng Y, Chauhan J (2020). Lineage-restricted regulation of SCD and fatty acid saturation by MITF controls melanoma phenotypic plasticity. Mol Cell.

[46] Wiemer AJ, Hegde S, Gumperz JE, Huttenlocher A (2011). A live imaging cell motility screen identifies prostaglandin E2 as a T cell stop signal antagonist. J Immunol.

[47] Cristescu R, Mogg R, Ayers M, Albright A, Murphy E, Yearley J, et al. Pan-tumor genomic biomarkers for PD-1 checkpoint blockade-based immunotherapy. Science. 2018;362:eaar3593. 10.1126/science.aar3593 PMC6718162 30309915

[48] Gunderstofte C, Iversen MB, Peri S, Thielke A, Balachandran S, Holm CK (2019). Nrf2 negatively regulates Type I interferon responses and increases susceptibility to herpes genital infection in mice. Front Immunol.

[49] Olagnier D, Brandtoft AM, Gunderstofte C, Villadsen NL, Krapp C, Thielke AL (2018). Nrf2 negatively regulates STING indicating a link between antiviral sensing and metabolic reprogramming. Nat Commun.

[50] Falahat R, Perez-Villarroel P, Mailloux AW, Zhu G, Pilon-Thomas S, Barber GN (2019). STING signaling in melanoma cells shapes antigenicity and can promote antitumor T-cell activity. Cancer Immunol Res.

[51] Marloye M, Lawler SE, Berger G (2019). Current patent and clinical status of stimulator of interferon genes (STING) agonists for cancer immunotherapy. Pharm Pat Anal.

[52] Markosyan N, Li J, Sun YH, Richman LP, Lin JH, Yan F (2019). Tumor cell-intrinsic EPHA2 suppresses anti-tumor immunity by regulating PTGS2 (COX-2). J Clin Investig.

