# Supplementary material for: The transcription factor NRF2 enhances melanoma malignancy by blocking differentiation and inducing COX2 expression
Source: Oncogene. 2020 Sep 25;39(44):6841–55. doi: 10.1038/s41388-020-01477-8 (PMC7605435; doi:10.1038/s41388-020-01477-8)
Supplement: Supplementary file 1 — Supplementary Material [file 41388_2020_1477_MOESM1_ESM.pdf]

Supplementary Material:

Supplementary Methods

Immunohistochemistry

Mouse tumors were fixed in 4% paraformaldehyde, embedded in paraffin and sectioned. Sections were deparaffinized, rehydrated, and stained with hematoxylin and eosin or processed for immunohistochemistry for CD3. Immunohistochemical staining for CD3 (SP7, rabbit monoclonal) from Abcam (Cambridge, UK), used in a dilution of 1:100, was performed using standard immunohistochemical methods. After peroxidase blocking and heat pretreatment with citrate buffer pH 6,0 in a steamer for 8 minutes, the primary antibody was incubated for 1 hour and detected with a 2-step polymer system (Super Vision 2) from DCS (Hamburg, Germany), HRP conjugated, followed by the manufacturer´s instructions.
For quantification slides were digitized by using VENTANA DP 200 slide scanner (Roche, Basel, Switzerland) and quantification was done by positive cell detection with QuPath open-source software platform [1].

Proliferation assay

Cells were seeded in duplicates or triplicates in 6-well plates and treated as indicated. On indicated days of treatment, cells were harvested by trypsinization, resuspended in PBS and counted using a Neubauer hemocytometer.

Stable transfection

To generate NRF2, MITF and ATF4 overexpression constructs, murine *Nfe2l2* and human *MITF* and *ATF4,* were amplified via PCR and cloned into the transposase vector pSB-ET-iE, which allows integration of the gene in the presence of the sleeping beauty transposase (encoded on pCMV (CAT)T7 SB100x) (Cloning primers see below):

Primer	sequence
murine_Nfe2l2_fwd	5`-GCGGCTAGCATGATGGACTTGGAGTTGCCA-3`
murine_Nfe2l2_rev	5`-GCGAGCGCTCTAGTTTTTCTTTGTATCTGGCT-3`
human_MITF_fwd	5`-GCGGCTAGCATGCAGTCCGAATCGGGGAT-3`
human_MITF_rev	5´-GCGAGCGCTCTAACAAGTGTGCTCCGTCTC-3´
human_ATF4_fwd	5`-GCGGCTAGCATGACCGAAATGAGCTTCCTG-3`
human_ATF4_rev	5-GCGAGCGCTCTAGGGGACCCTTTTCTTCC-3`

The doxycycline responsive T6 promoter drives expression of the target gene and EGFP, with EGFP being flanked by an IRES site. For transfection, Fugene HD transfection reagent (Promega, Fitchburg, Massachusetts, USA) or Lipofectamine 3000 (Invitrogen, Carlsbad, USA) was used according to the manufacturer’s protocol. Positive cells were selected with 1

µg/ml puromycin (Calbiochem/Merck, Darmstadt, Germany). The pSB-ET-iE-BRAF^{V600E} expression vector was described previously [2].

CellRox assay

Cells were seeded in triplicates in a 96-well plate and treated as indicated. Incubation of cells with 500 µM H_2O_2 for 30 minutes served as a positive control. CellRox assay was performed in triplicates according to the manufacturer's protocol (Invitrogen, Carlsbad, USA). Immediately after CellRox staining, images and quantifications were done using a deep red (660 nm) and Hoechst channel of the Operetta microscope (Perkin Elmer, Waltham, USA).

Tietze assay

GSH concentrations were determined by using the modified Tietze assay [3]. 2 x 10^5 cells per sample were seeded and treated as indicated. Whole cell pellets were reconstituted in 1x PBS and 5 % sulfosalicylic acid (SSA; Sigma-Aldrich, St Louis, USA) in GSH assay buffer (143 mM phosphate buffer, 6.3 mM EDTA, adjusted to pH 7.5) in a ratio of 1:3. To solubilize cells, samples were frozen (-80°C) and thawed for 3 times and subsequently centrifuged (12000 rpm, 4 °C, 15 min). For GSH determination, assay buffer with freshly added 0.34 mM NADPH (Biomol, Hamburg, Germany), 6 mM 5-5`-dithiobis-2-nitrobenzoic acid (Sigma-Aldrich, St Louis, USA), H_2O and cell pellet supernatant were added to a 1.5 ml cuvette (in a 7:1:1:0.5 ratio) and incubated for 20 minutes at room temperature. Defined GSH concentrations in the range between 10 µM and 320 µM were used to generate a standard curve. The reaction was started by adding 1 U/ml of glutathione reductase (GR; Sigma-Aldrich, St Louis, USA). GSH concentrations were determined by observing the rate of change in absorption at 412 nm in a spectrophotometer. Concentrations of GSH were determined in duplicates and calculated relative to known standard concentrations and cell number.

Luciferase assay

Cells were seeded in 12-well plates and pretreated for one day with indicated concentrations of doxycycline to induce MITF expression. The next day cells were transfected with 950 ng of the Tyr-Luc-200 luciferase reporter construct (promoter element of the tyrosinase gene containing 200 bp upstream of transcription start site) and 400 ng or 800 ng of pcDNA3.1-Nrf2 construct. Total concentration of transfected DNA was kept constant at 2 µg by adding appropriate amounts of empty pcDNA3.1 vector. As transfection control and for normalization 50 ng of pGL4.74[hRluc/TK] vector (pRenilla) was used. Transfection was done with Fugene HD transfection reagent (Promega, Fitchburg, USA) in a 5:2 ratio accordingly to manufacturer's protocol, while cells were constantly kept under doxycycline treatment. After 48 h, transfected cells were lysed and assayed with the Dual-Luciferase® Reporter Assay (Promega, Fitchburg,

USA) according to the manufacturer's protocol. Firefly and Renilla luciferase activity were measured with a microplate reader system (Tristar LB941, Berthold Technologies, Bad Wildbad, Germany).

RNA extraction, cDNA synthesis and RT-qPCR

RNA isolation of cell pellets was performed using TRIzol reagent (Invitrogen, Carlsbad, USA) according to the manufacturer's protocol. cDNA synthesis was conducted using the RevertAid First Strand cDNA Synthesis Kit (Fermentas, Carlsbad, USA) with hexamer primers according to the manufacturer's protocol. RT-qPCRs were performed and analyzed with the Mastercycler ep realplex (Eppendorf, Hamburg, Germany) using SYBR Green reagent. Gene expression was normalized to *Actb* or *RPS14* as housekeeping genes using the $\Delta\Delta$ct method. The sequences of the oligonucleotides are indicated in Supplementary Table 3.

siRNA transfection

Cells were transfected with 120 nM non-targeting siRNA (Sigma-Aldrich, St Louis, USA; SIC001) or siRNA directed against human *NFE2L2* (Ambion, Carlsbad, USA, AM16704; ID3347, ID107967) using XtremeGene siRNA transfection reagent (Roche, Basel, Switzerland) in accordance with the manufacturer's instructions. NRF2 sequences are detailed in Supplementary table 1. After 24 hours, cells were reseeded to new dishes for further experiments.

CRISPR/Cas9 mediated gene knockout

For CRISPR/Cas9 mediated gene knockout in human and murine melanoma cells, one gRNA construct for human *NFE2L2* and *ATF4* each and two gRNA constructs for murine *Nfe2l2* were cloned using the vector pU6-(BbsI)CBh-Cas9-T2A-mCherry (Addgene #64324) in addition to non-targeting controls (cloning primers see Supplementary table 2). UACC-62 were transfected with single gRNA constructs and #781 cells were simultaneously transfected with both murine *Nfe2l2* gRNA constructs. Human melanoma cell lines were transfected by using Fugene HD transfection reagent (Promega, Fitchburg, USA) and murine #781 cells were transfected using Lipofectamin 3000 reagent (Invitrogen, Carlsbad, USA). Single clones were picked for verification by sequencing of genomic DNA.

Immunofluorescence

Cells were washed with 1x PBS and fixed in 4 % paraformaldehyde (Sigma-Aldrich, St Louis, USA). After permeabilization for 10 minutes in 0.2 % Triton X-100/PBS, samples were washed once with 0.1 % Triton X-100/PBS (Roth, Karlsruhe, Germany) and twice with 1x PBS. Subsequently, cells were quenched in 100 mM glycerol/PBS (Roth, Karlsruhe, Germany) and blocked in 1 % BSA/PBS (Serva, Heidelberg, Germany) for 30 minutes. Then, cells were

incubated with primary antibody in 1 % BSA/PBS for 1 hour at room temperature (NRF2: ab62352, Abcam, Cambridge, UK), diluted 1:500 in PBS with 1% bovine serum albumin). After three PBS washes, secondary antibody incubation was carried out in the dark for 1 h at room temperature. The respective Alexa Fluor® antibody was diluted 1:500 (Alexa Fluor® 488 goat anti-mouse IgG A11001; Alexa Fluor® 594 goat anti-rabbit IgG A11037 of Life Technologies, Carlsbad, USA). After washing with PBS, nuclear counterstaining was performed by incubating with Hoechst 34580 (Invitrogen, Carlsbad, USA) (1:10000 in PBS) for 10 minutes. Afterwards, samples were washed with PBS and ddH_2O and coverslips were embedded with Mowiol-DABCO (Sigma-Aldrich, St Louis, USA) on object slides. Samples were determined by inverted fluorescent microscopy (Leica, Wetzlar, Germany).

LC/MS-Analysis

Extraction of lipids in tissue homogenate: Tissue samples were cut into small pieces and homogenized with Ultraturrax after addition of 49 vol. of H_2O. 270 µl homogenate were mixed with 30 µl 10% acetic acid and 300 µl n-butanol / methanol (3/1, v/v). After the addition of 300 µl heptane / ethyl acetate (3/1, v/v) and 280 µl 1% acetic acid, samples were mixed and centrifuged (2 min. max rpm in an Eppendorf centrifuge). The resulting upper phase was transferred into a new Eppendorf cup, the lower phase was reextracted with 400 µl heptane/ethyl acetate (3/1, v/v). Upper phases were combined and evaporated at 35 °C under a stream of N_2 gas.

LC/MS-Analysis: The equipment used for LC/MS analysis was a Thermo Scientific Dionex Ultimate 3000 UHPLC system hyphenated with a Q Exactive mass spectrometer (QE-MS) equipped with a HESI probe (Thermo Scientific, Bremen, Germany). For LC/MS analysis, the residues of the lipid extractions were dissolved in 100 µl of iPrOH. Chromatographic separation was achieved by applying 3 µl dissolved sample on a Acclaim RSLC 120 C8 (2.2 µm particles, 50 × 2.1 mm) (Thermo Scientific, Bremen, Germany), combined with a Javelin particle filter (Thermo Scientific, Bremen, Germany) and a Acclaim 120 C8 (5 µm particles, 10 × 2 mm) (Thermo Scientific, Bremen, Germany) precolumn using a linear gradient of mobile phase A (CH_3CN/H_2O/formic acid (10/89.9/0.1, v/v/v)) and mobile phase B (CH_3CN/2-propanol/H_2O/formic acid (45/45/9.9/0.1, v/v/v/v)). The column was kept at 40°C and the LC gradient program was 20% solvent B for 2 min, followed by a linear increase to 100% solvent B within 5 min, then maintaining 100% B for 27 min, then returning to 20% B in 1 min and 5 min 20% solvent B for column equilibration before each injection. The column temperature was set to 40°C, the flow rate was maintained at 350 µL/min. The eluent was directed to the ESI source of the QE-MS from 2.0 min to 29 min after sample injection. For MS analysis, the following MS scan parameters were used: Scan Type: Full MS in alternating pos./neg. mode; Runtime: 2 min - 29 min; Resolution: 70,000; AGC-Target: 3E6; Maximum Injection Time: 200

ms; Scan Range: 200 - 1500 m/z. HESI Source Parameters: Sheath gas flow rate: 30; Auxiliary gas flow rate: 10; Sweep gas flow rate: 3; Spray voltage: 2.5 kV in pos. mode and 3.6 kV in neg. mode; Capillary temperature: 320 °C; S-lens RF level: 55.0; Aux Gas Heater temperature: 120 °C. Peaks corresponding to the calculated lipid masses (MIM +/- H+ ± 2 mMU) were integrated using TraceFinder software (Thermo Scientific, Bremen, Germany). Specific peak areas were normalized to total lipid peak areas. Ultrapure water was obtained from a Millipore water purification system (Milli-Q Merck Millipore, Darmstadt, Germany). LC/MS solvents, LC/MS NH4OAc and standard compounds were purchased from Merck (Darmstadt, Germany).

RNA sequencing

RNA was isolated from triplicates using the RNeasy Kit (Qiagen, Venlo, Netherlands) with on-column DNase digestion according to the manufacturer's instruction. The quality of total RNA was determined using the Experion Automated Electrophoresis System (BioRad, Hercules, USA) or a Fragment analyzer (Agilent, Santa Clara, USA). 1 µg of total RNA was used for Poly-A+ RNA isolation using the NEBNext Poly(A) mRNA Magnetic Isolation Module (New England Biolabs, Ipswich, USA). NEBNext® Ultra™ II RNA Library Prep Kit for Illumina® (New England Biolabs, Ipswich, USA) was used for library preparation according to the manufacturer's instructions. cDNA libraries were amplified with 12 PCR cycles. Library quantities were determined with the Experion Automated Electrophoresis System (BioRad, Hercules, USA) or a Fragment analyzer (Agilent, Santa Clara, USA). Sequencing was done using a NextSeq500 Illumina (San Diego, USA) (NB500931) platform (single end sequencing, 75 cycles). Afterwards, all human data were aligned to the genome (Homo_sapiens.GRCh38.dna.toplevel.fa) using STAR [4]. Expected read counts for each gene were calculated using RSEM [5]. Differential expression was calculated by the Bioconductor/R package and DESeq2 software [https://bioconductor.org/packages/3.7/bioc/vignettes/DESeq2/inst/doc/DESeq2.html] [6], followed by gene set enrichment analysis (GSEA, BROAD Institute).

For NRF2 knock out experiments *in vitro* and in *in vivo*, the FASTQs were checked for quality using FASTQC (Babraham Bioinformatics) followed by alignment to mm10 mouse genome build using Bowtie2 (v2.3.4.1) [7], using -N 1 option. All aligned reads containing files were normalized to same reads depth using Samtools (v1.3). These read normalized files were then used for differential expression regulation analysis using edgeR protocol [8] implemented in R v3.5.2. Briefly, the reads from bam files were read into R using *readGAlignment* function, followed by extraction of read counts for every gene with summarize Overlaps function. Non expressed genes were removed, and dispersion was calculated followed by p- and q- value calculation using Benjamini Hochberg correction. All further graphs were generated using

ggplot2 (https://ggplot2.tidyverse.org). NGS data are available under the Bioproject accession number PRJNA601317 and GEO accession number GSE141912.

Chromatin immunoprecipitation (ChIP) and sequencing

After treatment with either the NRF2 stabilizer sulforaphane (SFN) (7.5 µM) or DMSO (1:1000) for 24 h, chromatin immunoprecipitation (ChIP) was done with 3.0 x 10^7 UACC-62 cells per sample as described previously [9]. To isolate NRF2-coupled chromatin fragments, 3 µg of NRF2 antibody ([EP1808Y], ab62532, abcam, Cambridge, UK) was used. For ChIP sequencing, purified ChIP-DNA was quantified using the Qubit® DNA quantification assay system (Thermo Scientific, Waltham, USA). 4 ng DNA were used for cDNA libraries preparation using the NEBNext® Ultra II DNA Library Prep Kit for Illumina® (New England Biolabs, Ipswich, USA) according to the manufacturer's instructions. DNA fragments were amplified by 12 cycles of PCR and library quality was analyzed with a Fragment Analyzer (Agilent, Santa Clara, USA). Sequencing was done using a NextSeq500 Illumina (San Diego, USA) (NB500931) platform. Afterwards, sequencing data were uploaded and processed by using the Galaxy web platform (usegalaxy.org; [10]).

ATF4 ChIP sequencing was performed in human 501mel melanoma cells as described [11], using rabbit polyclonal anti-ATF4 antibody (#11815, Cell Signalling Technology, Danvers, USA) and an input control.

References

1 Bankhead P, Loughrey MB, Fernandez JA, Dombrowski Y, McArt DG, Dunne PD *et al*. QuPath: Open source software for digital pathology image analysis. *Sci Rep* 2017; 7: 16878.

2 Maurus K, Hufnagel A, Geiger F, Graf S, Berking C, Heinemann A *et al*. The AP-1 transcription factor FOSL1 causes melanocyte reprogramming and transformation. *Oncogene* 2017; 36: 5110-5121.

3 Tietze F. Enzymic method for quantitative determination of nanogram amounts of total and oxidized glutathione: applications to mammalian blood and other tissues. *Anal Biochem* 1969; 27: 502-522.

4 Dobin A, Davis CA, Schlesinger F, Drenkow J, Zaleski C, Jha S *et al*. STAR: ultrafast universal RNA-seq aligner. *Bioinformatics* 2013; 29: 15-21.

5 Li B, Dewey CN. RSEM: accurate transcript quantification from RNA-Seq data with or without a reference genome. *BMC Bioinformatics* 2011; 12: 323.

6 Love MI, Huber W, Anders S. Moderated estimation of fold change and dispersion for RNA-seq data with DESeq2. *Genome Biol* 2014; 15: 550.

7 Langmead B, Salzberg SL. Fast gapped-read alignment with Bowtie 2. *Nat Methods* 2012; 9: 357-359.

8 Robinson MD, McCarthy DJ, Smyth GK. edgeR: a Bioconductor package for differential expression analysis of digital gene expression data. *Bioinformatics* 2010; 26: 139-140.

9 Pattschull G, Walz S, Grundl M, Schwab M, Ruhl E, Baluapuri A *et al*. The Myb-MuvB Complex Is Required for YAP-Dependent Transcription of Mitotic Genes. *Cell Rep* 2019; 27: 3533-3546 e3537.

10 Afgan E, Baker D, Batut B, van den Beek M, Bouvier D, Cech M *et al*. The Galaxy platform for accessible, reproducible and collaborative biomedical analyses: 2018 update. *Nucleic Acids Res* 2018; 46: W537-W544.

11 Louphrasitthiphol P, Siddaway R, Loffreda A, Pogenberg V, Friedrichsen H, Schepsky A *et al*. Tuning Transcription Factor Availability through Acetylation-Mediated Genomic Redistribution. *Mol Cell* 2020.

12 Riesenberg S, Groetchen A, Siddaway R, Bald T, Reinhardt J, Smorra D *et al*. MITF and c-Jun antagonism interconnects melanoma dedifferentiation with pro-inflammatory cytokine responsiveness and myeloid cell recruitment. *Nat Commun* 2015; 6: 8755.

13 Laurette P, Strub T, Koludrovic D, Keime C, Le Gras S, Seberg H *et al*. Transcription factor MITF and remodeller BRG1 define chromatin organisation at regulatory elements in melanoma cells. *Elife* 2015; 4.

Supplementary Figure Legends:

Supplementary Figure 1: Effects of NRF2 knockdown in human melanoma cells

A: Upper image: Protein blot of NRF2 after siRNA mediated *NFE2L2* knockdown with two independent siRNAs. Actin or tubulin served as loading control. Lower image: corresponding real-time PCR, derived from 2-3 experiments.

B: Heatmap, showing the expression of the HALLMARK gene set "reactive oxygen species pathway" in UACC-62 cells transfected with non-targeting or *NFE2L2*-specific siRNA (N2), each in triplicates.

Supplementary Figure 2: Most deregulated pathways in *NFE2L2* knockdown melanoma cells

A: Functional characteristics of altered transcripts in UACC-62 melanoma cells transfected with control versus *NFE2L2*-specific siRNA (N2), as revealed by GSEA analysis (KEGG pathways). Shown are the top three up- and downregulated KEGG pathways. NES: Normalized enrichment score. **B-C**: Real-time PCR of genes from the categories cell cycle/replication **(B)** and oxidative phosphorylation/citric acid cycle (mitochondrial genes) **(C)**, using two independent siRNAs against *NFE2L2* (N1, N2). Relative expression levels referred to control siRNA are shown (dotted line). Two-tailed Student's t test was carried out to calculate significant differences between each *NFE2L2*-specific siRNA and the control siRNA. (*$p<0.05$, **$p<0.01$, ***$p<0.001$). Error bars represent SD. Experiments were done 3-4 times.

Supplementary Figure 3: COX2 regulation by NRF2

A: Immunoblot of NRF2 and COX2 in A375 cells after a combination of H_2O_2 treatment (400 µM, 5 h) and siRNA mediated *NFE2L2* knockdown. Actin served as loading control. **B:** Corresponding real-time PCR of *PTGS2*. The experiment was done twice. **C:** Proliferation of control and NRF2-ko UACC-62 cells. Cells were counted after 5 days. The experiments were carried out in triplicates. **D:** Real-time PCR of indicated cell cycle genes in control and NRF2-knockout UACC-62 cells. The experiment was done 3 times. **E:** Real-time PCR of indicated genes in control and NRF2-knockout UACC-62 cells after H_2O_2 treatment (400 µM, 5 h). The experiment was done at least 3 times. Two-way ANOVA with Tukey's multiple comparisons posttest was carried out (*$p<0.05$, **$p<0.01$, ***$p<0.001$). Error bars represent SD. **F:** PGE2 secretion in UACC-62 cells expressing Dox-inducible NRF2 (1000 ng/ml, 2 d) and tBHQ (10 µM, 2 d). ELISA assay was done in duplicates.

Supplementary Figure 4: Inverse regulation of *PTGS2* and *MITF* by TNFα

A: Genome browser tracks of promotor regions of *PTGS2* (left) and *HMOX1* loci (right) with NRF2 binding, evaluated by ChIP-Seq analysis. Where indicated, NRF2 was stabilized by treatment with sulforaphane (SFN). **B:** Immunoblot of NRF2 and COX2 in UACC-62 wt and NRF2-ko cells after TNFα treatment (50 ng/ml, 12 h). Actin served as loading control. **C:** RNA expression of *PTGS2* and *MITF* in MZ7 melanoma cells after TNFα treatment (1000 U/ml, 24 h). Data are extracted from GSE71798 [12].

Supplementary Figure 5: Effect of NRF2 on MITF target genes

A: Gene ontology (GO) term analysis of biological processes induced after knockdown of *NFE2L2* (N2) in UACC-62 cells (analyzed by DAVID functional annotation tool, https://david.ncifcrf.gov). **B:** Real-time PCR of genes from the category "pigmentation", using two independent siRNAs against *NFE2L2* (N1, N2). Relative expression levels referred to control siRNA are shown (dotted line). Two-tailed Student's t test was carried out to calculate significant differences between each *NFE2L2*-specific siRNA and the control siRNA. (*p<0.05, ***p<0.001). Error bars represent SD. Experiments were done 3-4 times. **C:** Protein expression of MITF and MLANA in UACC-62 control and NRF2-ko cells. Vinculin served as loading control. **D:** Genome browser tracks of promotor regions of *MITF* with NRF2 binding, evaluated by ChIP-Seq analysis. **E:** Protein blot of MITF and NRF2 in melanoma cell lines with low or high MITF status ($MITF^{low}$ and $MITF^{high}$, respectively), representing the general differentiation state. Actin served as loading control. **F:** Real-time PCR of *TYR*, *DCT* and *MLANA* in UACC-62 and M14 cells after H_2O_2 (400 µM, 5 h) or TNFα treatment (50 ng/ml, 24 h). The experiment was done 2-3 times. Data are related to the respective untreated control, which was set as 1 (dotted line). **G:** Real-time PCR of *TYR*, *DCT* and *MLANA* in UACC-62 control and NRF2-ko cells after TNFα treatment (50 ng/ml) for 1 or 3 days. The experiment was done 4 times. Data are related to the respective untreated control, which was set as 1. Two-tailed Student's t test was carried out to calculate significant differences between 1 d and 3 d of TNFα treatment. (*p<0.05, **p<0.01, ***p<0.001). Error bars represent SD. **H:** RNA expression of *PTGS2* in MZ7 melanoma cells (left) and 501mel melanoma cells (right) after siRNA mediated knockdown of *MITF*. Data are extracted from GSE71798 and GSE61966, respectively [12, 13]. **I:** Immunoblot of NRF2 and COX2 after *NFE2L2* knockdown in A549 cells. Actin served as loading control.

Supplementary Figure 6: Establishment of murine NRF2-knockout melanoma cells

A: Schematic overview of *Nfe2l2* exons in the mouse genome and the positions of used murine CRISPR/Cas9 guide RNAs. **B:** Immunoblot of NRF2 and COX2 in two independent sets of wt and NRF2-ko murine melanoma cells (cell line #781) after H_2O_2 treatment (400 µM, 5 h). Actin

served as loading control. **C:** Corresponding real-time PCR of indicated genes. *Hmox1*, *Slc7a11* and *Nqo1* are classical NRF2 target genes and served as control. Data were derived from at least three experiments, and two-way ANOVA with Sidak's multiple comparisons posttest was carried out (*p<0.05, **p<0.01, ***p<0.001). Error bars represent SD. **D:** MTT viability assay of indicated wt or NRF2-ko #781 cells after H_2O_2 treatment (25 µM, 24 h). The experiment was done once in triplicates.

Supplementary Figure 7: Transcriptional effect of NRF2 knockout in tumors in vivo
A: Functional characteristics of altered transcripts in *Nfe2l2* knockout tumors, as revealed by GSEA analysis (Hallmarks). NES: Normalized enrichment score. **B:** Relative expression of cytotoxic T cell markers in tumors derived from #781 control (wt-1) and *Nfe2l2* knockout cells (ko-1), as measured by real-time PCR with all available tumors. Significance was calculated with two-tailed Student's t test (*p<0.05, **p<0.01, ***p<0.001). **C:** Quantification of CD3+ T cell infiltration into #781 control (n=6) and NRF2 ko tumors (n=6) done with positive cell detection of QuPath open-source software [1]. After vector stain estimation for each slide, whole tumors, excluding necrotic parts, were quantified for positive nuclear DAB staining. **D:** Corresponding representative images. Brown staining indicates CD3 signal. **E:** Infiltration of indicated immune cell populations into SKCM melanomas in relation to *IFIH1* expression, as determined by the Tumor Immune Estimation Resource TIMER (https:://cistrome.shinyapps.io/timer/). Error bars represent SD.

Supplementary Figure 1:

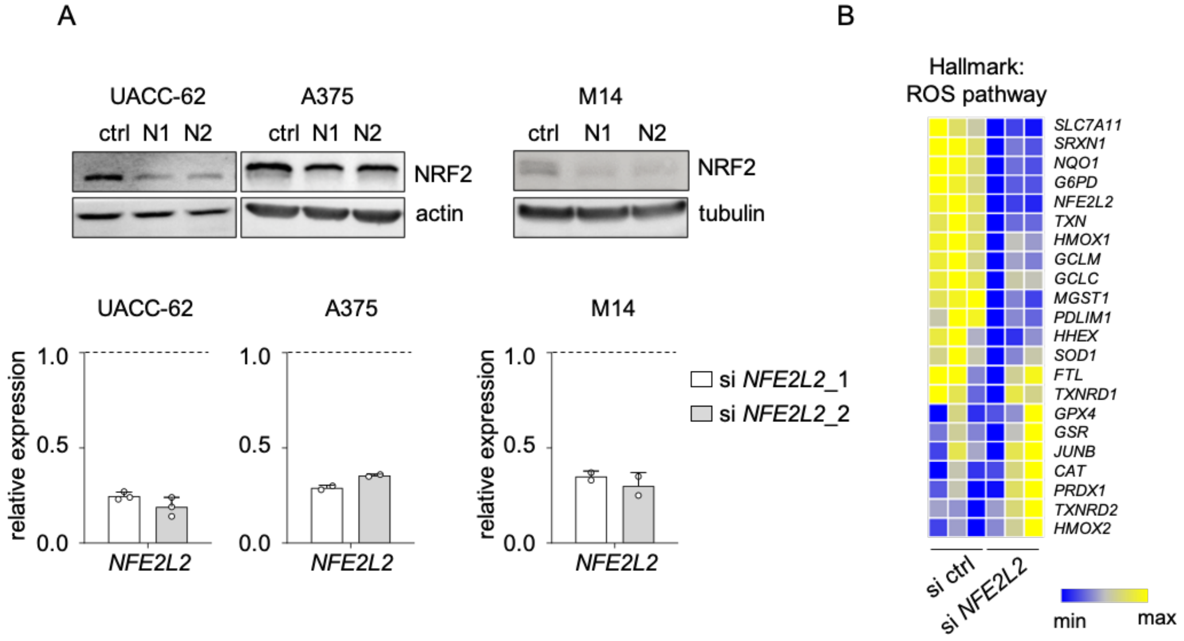

Supplementary Figure 2:

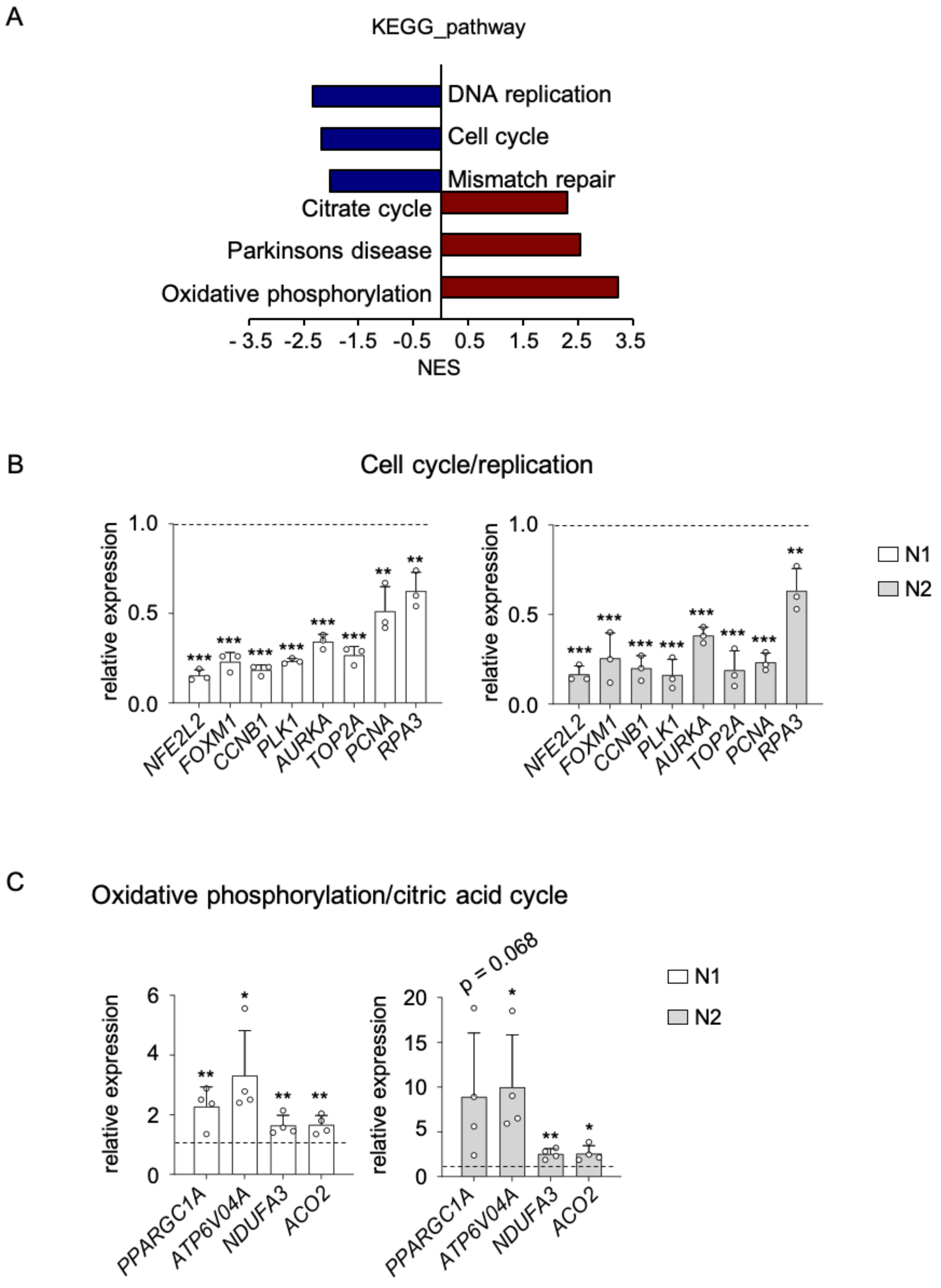

Supplementary Figure 3:

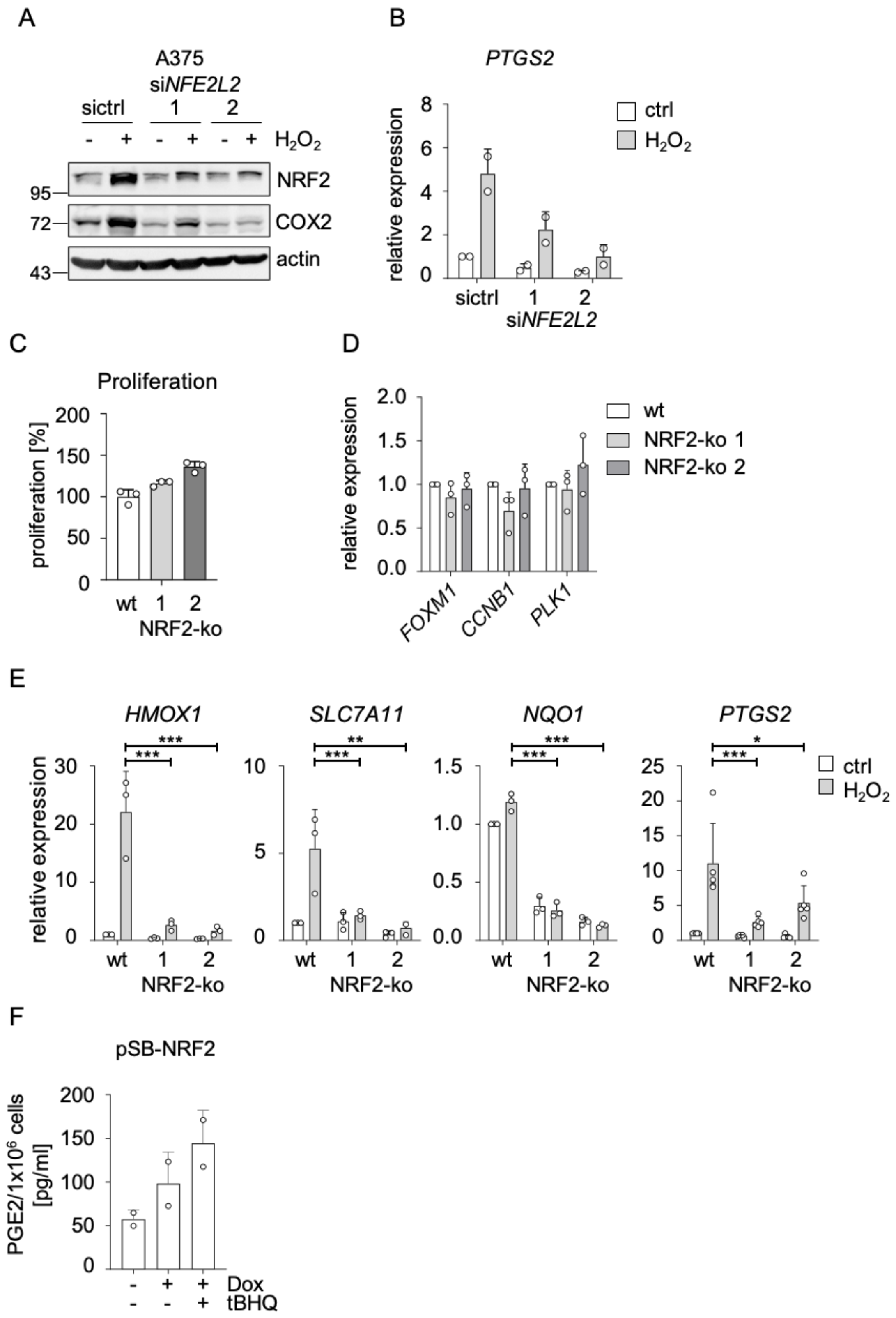

Supplementary Figure 4:

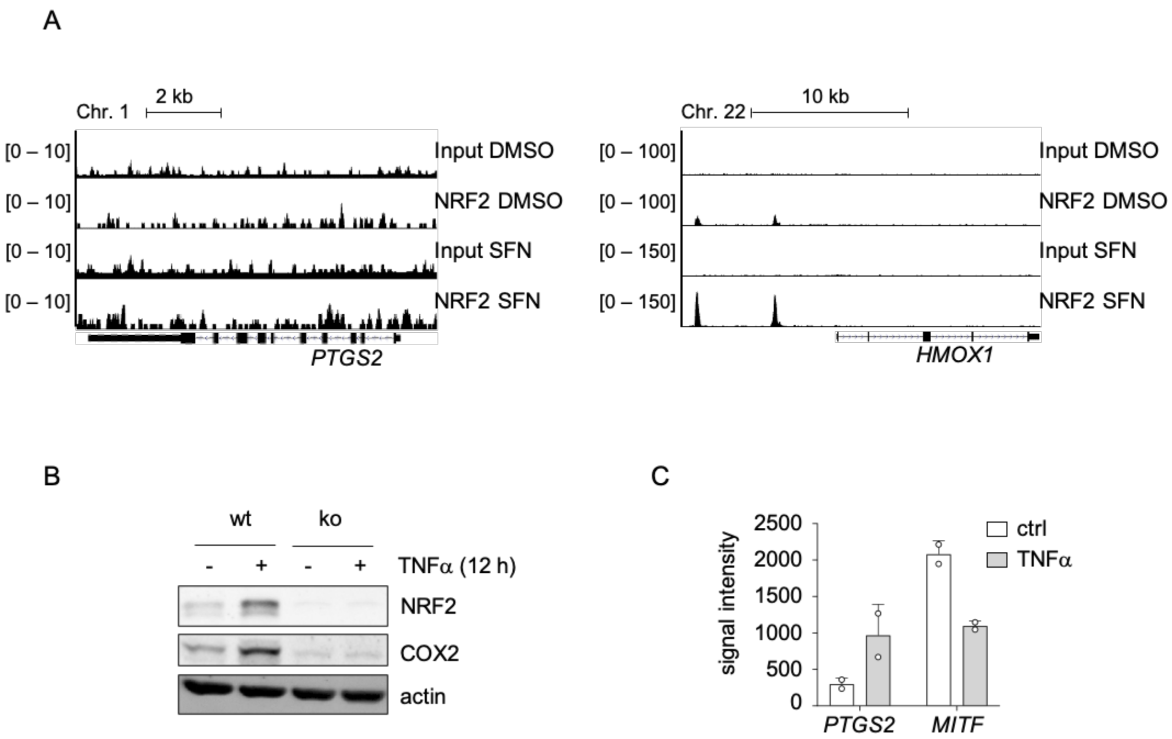

Supplementary Figure 5

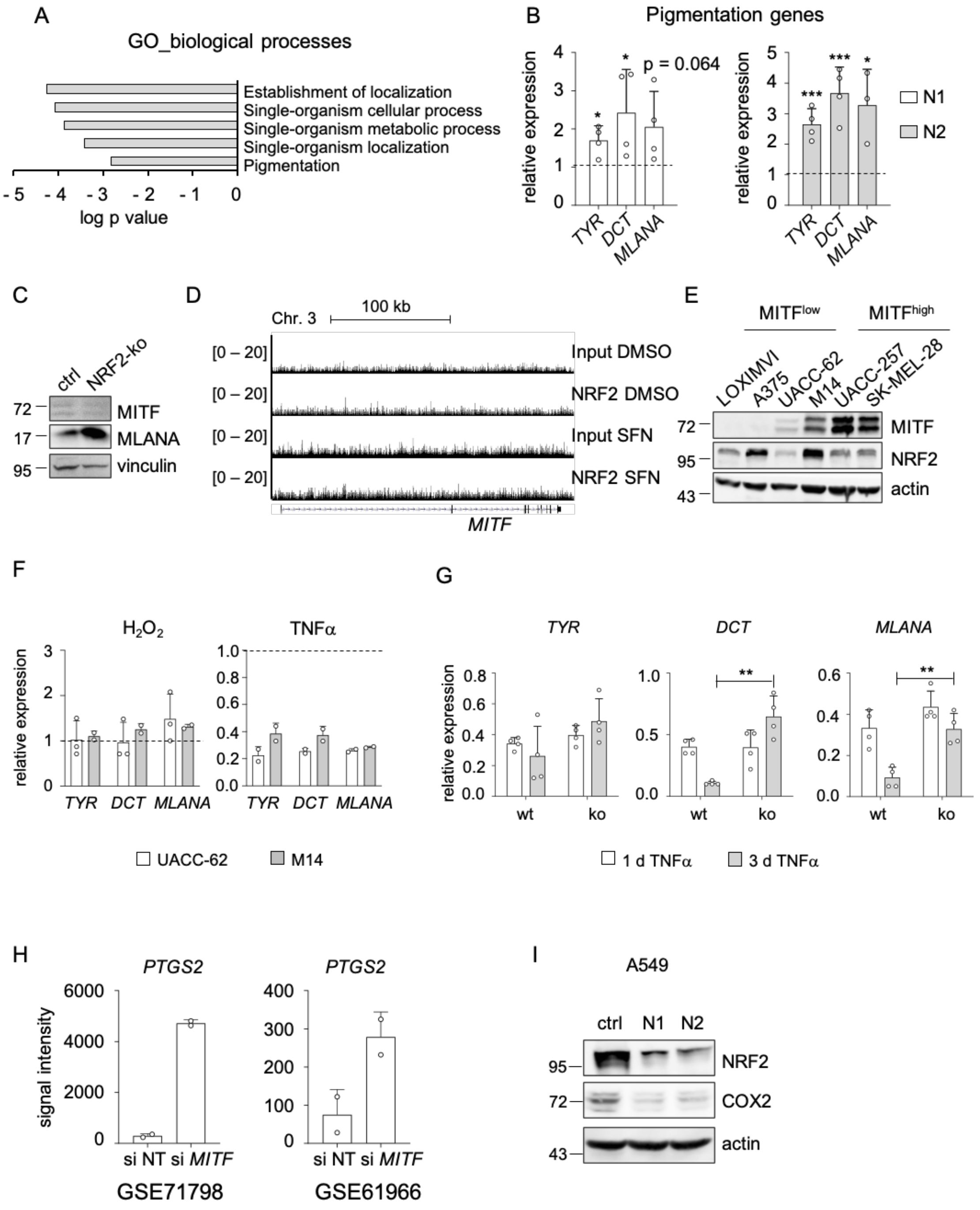

Supplementary Figure 6:

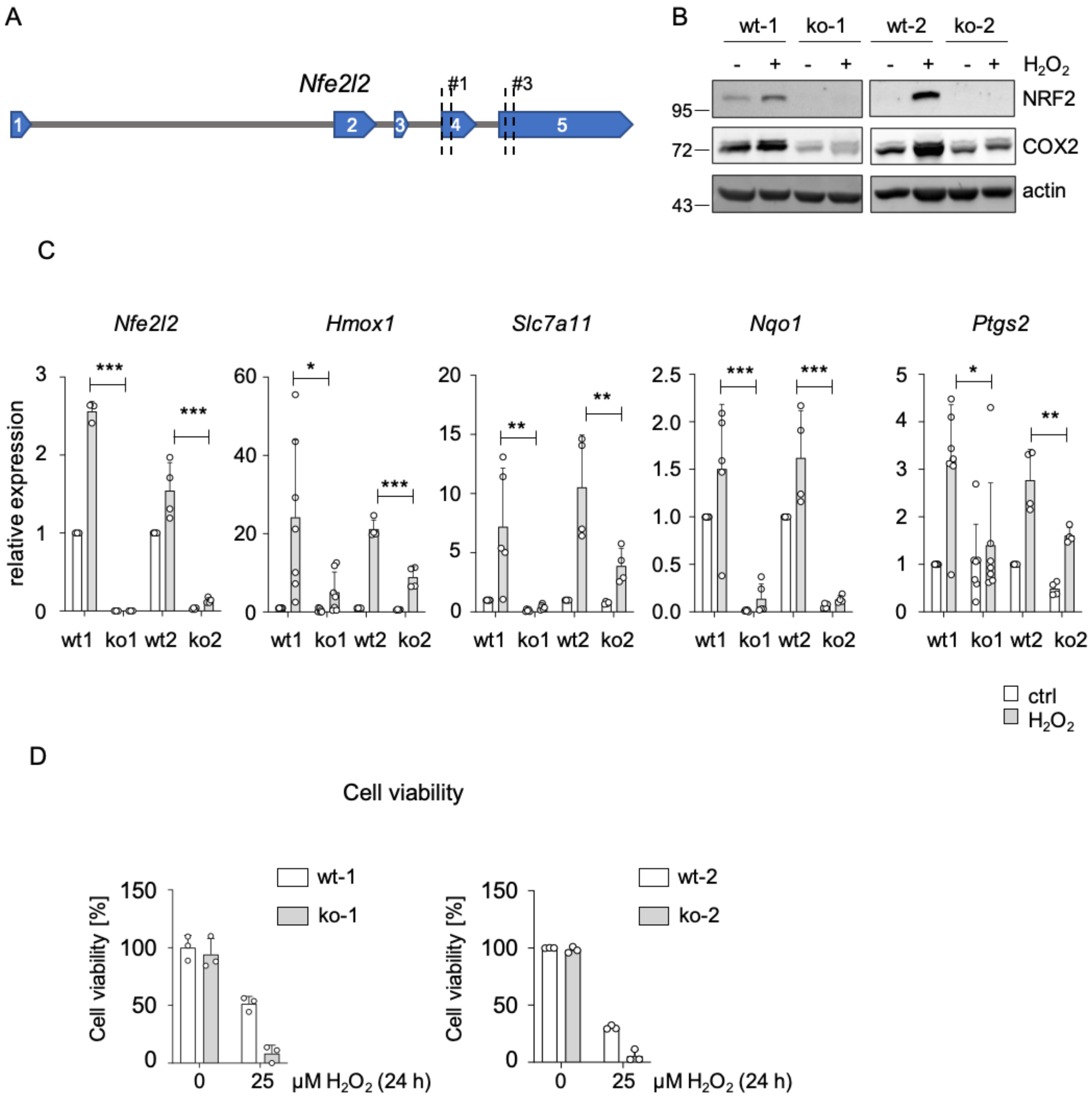

Supplementary Figure 7

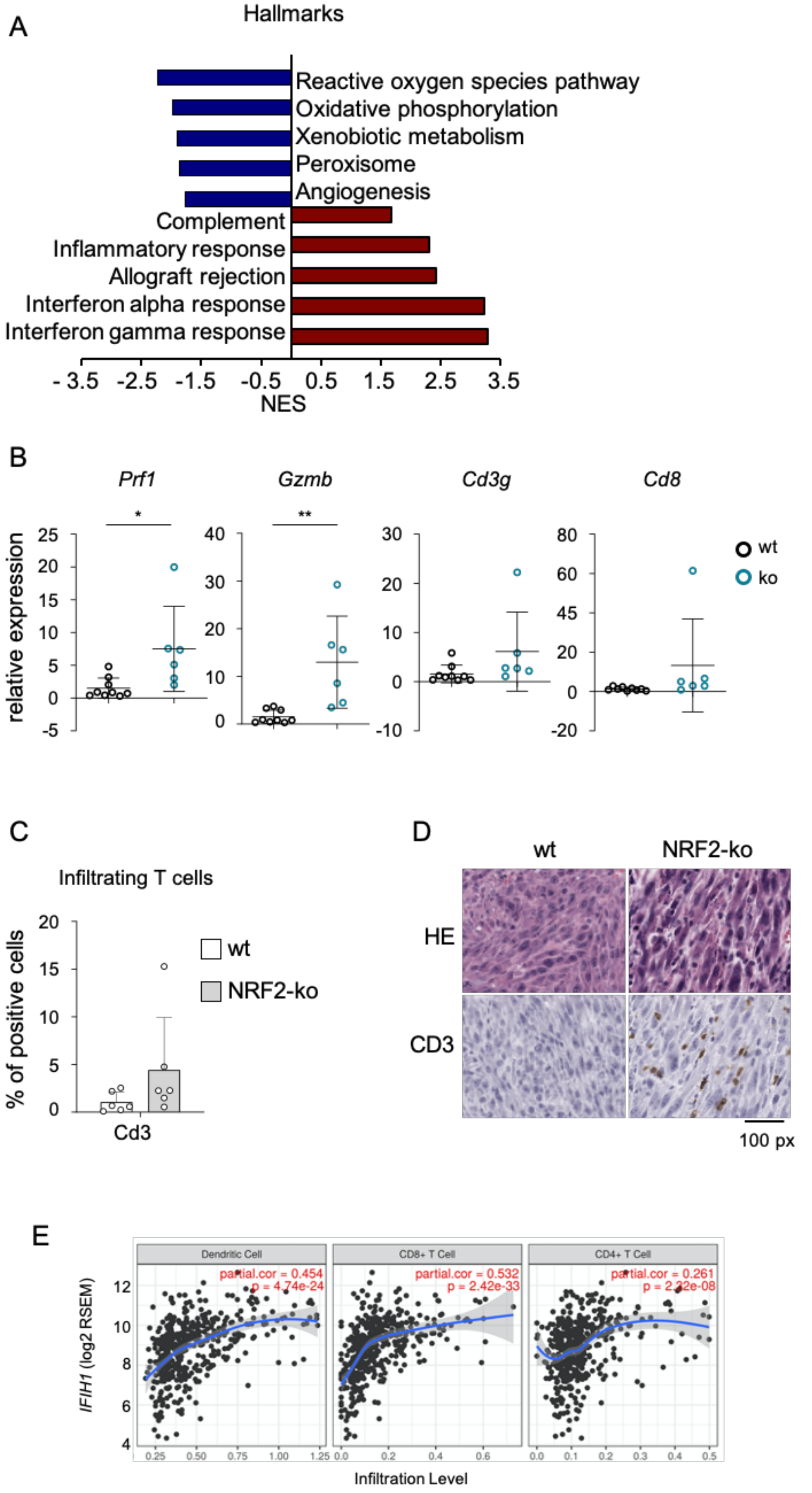

Supplementary Table 1:

siRNA sequences used in this study

siRNA	Target sequence	Identifier (Ambion, Carlsbad, USA)
si*NFE2L2*_1	5'-GGAGAAAAUGACAAAAGC-3'	ID3347
si*NFE2L2*_2	5'-GGAGCUAUUAUCCAUUCCU-3'	ID107967

Supplementary Table 2:

gRNA cloning sequences used in this study

gRNA	upstream cloning primer	downstream cloning primer
*gRNA_Nfe2l2*_1 (mouse)	5`-CACCGTGAAGACTGAACTTTCAGCG-3`	5`-AAACCGCTGAAAGTTCAGTCTTCAC-3`
*gRNA_Nfe2l2*_3 (mouse)	5`-CACCGTCCTCGCTGGAAAAAGAAGT-3`	5`-AAACACTTCTTTTTCCAGCGAGGAC-3`
gRNA_non-targeting (mouse)	5`-CACCGGTATTACTGATATTGGTGGG-3`	5`-AAACCCCACCAATATCAGTAATAGC-3`
gRNA_NFE2L2 (human)	5`-CACCGCATACCGTCTAAATCAACAG-3`	5`-AAACCTGTTGATTTAGACGGTATGC-3`
gRNA_ATF4	5`-CACCGTAATAAGCAGCCCCCCCAGA-3`	5`-AAACTCTGGGGGGGCTGCTTATTAC-3`
gRNA_non-targeting (human)	5`-CACCGGCGAGGTATTCGGCTCCGCG-3`	5`-AAACCGCGGAGCCGAATACCTCGCC-3`

Supplementary Table 3:

Oligonucleotides used for RT-qPCR

**Human genes**		
**Gene**	**Forward primer**	**Reverse primer**
ACO2	5‘-TCCCAGTTCACCATCACTCC-3‘	5‘-ATGTCCTTCCTGTCCCACTG-3‘
ACTB	5‘-GGCATCCTGACCCTGAAGTA-3‘	5‘-GGGGTGTTGAAGGTCTCAAA-3‘
ATP6V04A	5‘-AGCCCCTCCCACATTTAACA-3‘	5‘-CATCACAGCGAACAGGAAGG-3‘
AURKA	5‘-AATGATTGAAGGTCGGATGC-3‘	5‘-CCTGGCTCCCTCTGTTACAA-3‘
CCNB1	5‘-CGGGAAGTCACTGGAAACAT-3‘	5‘-AAACATGGCAGTGACACCAA-3‘
DCT	5‘-GGTTCCTTTCTTCCCTCCAG-3‘	5‘-AACCAAAGCCACCAGTGTTC-3‘
EGFR	5‘-AGTGCTGGATGATAGACGCA-3‘	5‘-CCTGAATGACAAGGTAGCGC-3‘
FOXM1	5‘-ACCCAAACCAGCTATGATGC-3‘	5‘-GAAGCCACTGGATGTTGGAT-3‘
HMOX1	5‘-CTTCTTCACCTTCCCCAACA-3‘	5‘-CTTCTTCACCTTCCCCAACA-3‘
IFIH1	5‘-TCCAACTGCTGAACCTCCTT-3‘	5‘-GCAATCCGGTTTCTGTCTTC-3‘
IFIT1	5‘-GCAGCCAAGTTTTACCGAAG-3‘	5‘-GCCCTATCTGGTGATGCAGT-3‘
MAFF	5‘-GAGAGCTGAGCGAGAACACG-3‘	5‘-CGTAGCCACGGTTTTTGAGT-3‘
MITF	5‘-GGGCTTGATGGATCCTGCTT-3‘	5‘-GCCAGTGCTCTTGCTTCAGA-3‘
MLANA	5‘-GCTCATCGGCTGTTGGTATT-3‘	5‘-GGGAACCACAGGTTCACAGT-3‘
NDUFA3	5‘-GGGGCCTCGCTGTAATTCTG-3‘	5‘-GACGGGCACTGGGTAGTTG-3‘
NFE2L2	5‘-GAGAGCCCAGTCTTCATTGC-3‘	5‘-GTTTGGCTTCTGGACTTGGA-3‘
NQO1	5‘-AGCCCAGATATTGTGGCTGA-3‘	5‘-CGGAAGGGTCCTTTGTCATA-3‘
PCNA	5‘-TGGAGAACTTGGAAATGGAAA-3‘	5‘-GAACTGGTTCATTCATCTCTATGG-3‘
PLK1	5‘-AAGATCTGGAGGTGAAAATAGGG-3‘	5‘-AGGAGTCCCACACAGGGTCT-3‘
PPARGC1A	5‘-TCAGTACCCAGAACCATGCA-3‘	5‘-GGGACGTCTTTGTGGCTTTT-3‘
PTGS2	5‘-TGAAACCCACTCCAAACACA-3‘	5‘-GAGAAGGCTTCCCAGCTTTT-3‘
RPA3	5‘-AAGCCTGTCTGCTTCGTAGG-3‘	5‘-AAGCCTGTCTGCTTCGTAGG-3‘
RPS14	5‘-CTCAGGTGGCTGAAGGAGAG-3‘	5‘-GCAGCCAACATAGCAGCATA-3‘
RSAD2	5‘-GGGAGAGGTGGTTCCAGAAT-3‘	5‘-ACCACCTCCTCAGCTTTTGA-3‘
SLC7A11	5‘-TTTGCACCCTTTGACAATGA-3‘	5‘-GGAAAACAAAGCTGGGATGA-3‘
TOP2A	5‘-AATCTCAGAGCTTCCCGTCA-3‘	5‘-TGCCTCTGCCAGTTTTTCTT-3‘
TYR	5‘-CCGCTATCCCAGTAAGTGGA-3‘	5‘-TACGGCGTAATCCTGGAAAC-3‘
**Murine genes**		
**Gene**	**Forward primer**	**Reverse primer**
Actb	5‘-GCTACAGCTTCACCACCACA-3‘	5‘-AAGGAAGGCTGGAAAAGAGC-3‘
Cd3g	5‘-GACTTGTGGCTTGACTGACA-3‘	5‘-CTCGAGGGTCTTTGGCATTG-3‘
Cd8a	5‘-TCAGTTCTGTCGTGCCAGTC-3‘	5‘-GCCGACAATCTTCTGGTCTC-3‘
Gzmb	5‘-GCATTCCCCACCCAGACTAT-3‘	5‘-GCTTCACATTGACATTGCGC-3‘
Hmox1	5‘-CACGCATATACCCGCTACCT-3‘	5‘-CCAGAGTGTTCATTCGAGCA-3‘
Ifih1	5‘-TCACTGATCTGCCCTCTCCT-3‘	5‘-CCTTCTCGAAGCAAGTGTCC-3‘
Ifit1	5‘-ATGGGAGAGAATGCTGATGG-3‘	5‘-AGGAACTGGACCTGCTCTGA-3‘
Isg15	5‘-AAGAAGCAGATTGCCCAGAA-3‘	5‘-TCGCTGCAGTTCTGTACCAC-3‘
Maff	5‘-GCGAGTTGAGCGAGAACAC-3‘	5‘-GTAGCCGCGGTTCTTGAGT-3‘
Nfe2l2	5‘-AGGACATGGAGCAAGTTTGG-3‘	5‘-TTCTTTTTCCAGCGAGGAGA-3‘
Nqo1	5‘-CTGGCCCATTCAGAGAAGAC-3‘	5‘-GTCTGCAGCTTCCAGCTTCT-3‘
Prf1	5‘-CGCATGTACAGTTTTCGCCT-3‘	5‘-TGGTAAGCATGCTCTGTGGA-3‘
Ptgs2	5‘-GGCCATGGAGTGGACTTAAA-3‘	5‘-ACCTCTCCACCAATGACCTG-3‘

Rsad2	5‘-AAGGTTTTCCAGTGCCTCCT-3‘	5‘-ATTCAGGCACCAAACAGGAC-3‘
Slc7a11	5‘-TCCACAAGCACACTCCTCTG-3‘	5‘-TGCATATCTGGGCGTTTGTA-3‘
Tmem173	5‘-GGCATCAAGAATCGGGTTTA-3‘	5‘-ATCCTGTGACATGGCAAACA-3‘
